# *UMOD* Genotype-Blinded Trial of Ambulatory Blood Pressure Response to Torasemide

**DOI:** 10.1161/HYPERTENSIONAHA.124.23122

**Published:** 2024-07-30

**Authors:** Linsay McCallum, Stefanie Lip, Alex McConnachie, Katriona Brooksbank, Iain M. MacIntyre, Alexander Doney, Andrea Llano, Alisha Aman, Thomas M. Caparrotta, Gareth Ingram, Isla S. Mackenzie, Anna F. Dominiczak, Thomas M. MacDonald, David J. Webb, Sandosh Padmanabhan

**Affiliations:** 1 Queen Elizabeth University Hospital, Glasgow, Scotland, United Kingdom (L.M.C., S.L., A.L., G.I., S.P.); 2 School of Cardiovascular and Metabolic Health (L.M.C., S.L., K.B., A.A., A.F.D., S.P.), University of Glasgow, Scotland, United Kingdom.; 3 Robertson Centre for Biostatistics, School of Health and Wellbeing (A.M.C.), University of Glasgow, Scotland, United Kingdom.; 4 Clinical Pharmacology Unit and Research Centre, University of Edinburgh/BHF Centre of Research Excellence, United Kingdom (I.M.I., T.M.C., D.J.W.); 5 MEMO Research, University of Dundee, Ninewells Hospital and Medical School, United Kingdom (A.D., I.S.M., T.M.M.D.).

**Keywords:** blood pressure, genotype, hypertension, torsemide, uromodulin

## Abstract

**BACKGROUND:**

UMOD (uromodulin) has been linked to hypertension through potential activation of Na^+^-K^+^-2Cl^−^ cotransporter (NKCC2), a target of loop diuretics. We posited that hypertensive patients carrying the rs13333226-AA *UMOD* genotype would demonstrate greater blood pressure responses to loop diuretics, potentially mediated by this UMOD/NKCC2 interaction.

**METHODS:**

This prospective, multicenter, genotype-blinded trial evaluated torasemide (torsemide) efficacy on systolic blood pressure (SBP) reduction over 16 weeks in nondiabetic, hypertensive participants uncontrolled on ≥1 nondiuretic antihypertensive for >3 months. The primary end point was the change in 24-hour ambulatory SBP (ABPM SBP) and SBP response trajectories between baseline and 16 weeks by genotype (AA versus AG/GG) due to nonrandomized groups at baseline (ClinicalTrials.gov: NCT03354897).

**RESULTS:**

Of 251 enrolled participants, 222 received torasemide and 174 demonstrated satisfactory treatment adherence and had genotype data. The study participants were middle-aged (59±11 years), predominantly male (62%), obese (body mass index, 32±7 kg/m^2^), with normal eGFR (92±17 mL/min/1.73 m²) and an average baseline ABPM of 138/81 mm Hg. Significant reductions in mean ABPM SBP were observed in both groups after 16 weeks (AA, −6.57 mm Hg [95% CI, −8.44 to −4.69]; *P*<0.0001; AG/GG, −3.22 [95% CI, −5.93 to −0.51]; *P*=0.021). The change in mean ABPM SBP (baseline to 16 weeks) showed a difference of −3.35 mm Hg ([95% CI, −6.64 to −0.05]; *P*=0.048) AA versus AG/GG genotypes. The AG/GG group displayed a rebound in SBP from 8 weeks, differing from the consistent decrease in the AA group (*P*=0.004 for difference in trajectories).

**CONCLUSIONS:**

Our results confirm a plausible interaction between UMOD and NKCC2 and suggest a potential role for genotype-guided use of loop diuretics in hypertension management.

**REGISTRATION:**

URL: https://www.clinicaltrials.gov; Unique identifier: NCT03354897.

**NOVELTY AND RELEVANCE**

**What Is New?**

- This is the first clinical trial of a genome-wide association study single nucleotide polyphormism and a novel pathway for hypertension mediated by the *UMOD* gene.
- Uncontrolled hypertensive patients with the uromodulin increasing AA genotype respond more significantly to the loop diuretic torasemide compared with AG/GG genotype carriers.
- Genotype-specific fall-rebound patterns in blood pressure response over a 16-week period are more prominent in the AG/GG carriers.

**What Is Relevant?**

- Provides evidence supporting the use of genotype-guided loop diuretics in managing uncontrolled hypertension.
- Highlights the need for further investigation into the role of uromodulin and its interaction with Na^+^-K^+^-2Cl^−^ cotransporter in blood pressure regulation.

**Clinical/Pathophysiological Implications?**

- Genotype-guided therapy could lead to more effective and tailored treatment strategies for hypertensive patients.

Pharmacotherapy using antihypertensive drug classes to reduce blood pressure (BP) is the mainstay of hypertension management, but only 1 in 5 patients have controlled hypertension, and hypertension-related cardiovascular death rates have increased over the past decade against previously declining trends.^1–3^ New antihypertensive drug development has stalled over the last 2 decades, and there has been little progress in individually targeted therapy by leveraging genomic and molecular information, despite evidence that this may be useful.^4,5^ There is accruing evidence from genome-wide association studies (GWAS) and transgenic experiments indicating that UMOD (uromodulin), encoded by the *UMOD* gene and exclusively expressed in the thick ascending limb of the loop of Henle and in the early part of the distal convoluted tubule, is involved in BP regulation through a putative interaction with the Na^+^-K^+^-2Cl^−^ cotransporter (NKCC2).^6–12^ GWAS show that the major A allele (with a frequency of 0.82) of the *UMOD* promoter single nucleotide polyphormism (SNP), rs13333226, is associated with a higher risk of hypertension,^9^ higher systolic and diastolic BP,^9,13,14^ lower estimated glomerular filtration rate (eGFR),^15^ increased urinary uromodulin excretion as well as renal *UMOD* expression,^8,9^ and higher fractional excretion of endogenous lithium.^9^ Mendelian randomization studies support a causal role for uromodulin on BP, independent of its effect on eGFR.^10^ *UMOD* gene knockout mice show a shift-to-left of the pressure-natriuresis curve,^7^ while mice overexpressing uromodulin show increased phosphorylation and consequent activation of NKCC2,^12^ upregulation of other distal electrolyte transporters along with downregulation of cyclooxygenase-2 and renin.^6^ Further support for an interaction between uromodulin and NKCC2 comes from studies that blocked NKCC2 with loop diuretics, its specific inhibitor. Treatment with furosemide, a loop diuretic, significantly enhanced natriuresis and reduced BP levels both in the transgenic mice overexpressing uromodulin and in a small retrospective analysis of treatment-naïve hypertensive individuals homozygous for the uromodulin increasing A allele.^12^ These data warrant the need for a randomized controlled trial to establish if hypertensive patients carrying the rs13333226-AA *UMOD* genotype would exhibit greater BP responses to loop diuretics, thereby reinforcing the hypothesis of an interaction between uromodulin and NKCC2 in regulating BP.

Our primary objective was to test whether hypertensive participants with uncontrolled BP homozygous for the uromodulin increasing genotype (rs13333226 AA) will show greater BP reduction with loop diuretics compared with those with the AG and GG genotypes.^16^ A positive result from this study would strengthen the case for a UMOD-NKCC2 BP pathway as well as support genotype-directed early use of loop diuretics for patients with hypertension.

## METHODS

### Data Availability

The data that support the findings of this study are available from the corresponding author upon reasonable request.

### Study Design and Participants

The protocol has been published.^16^ In this 16-week genotype-blinded multicenter trial, participants were identified and enrolled from secondary care BP clinics and general practices and from 3 sites in Scotland. Home BP monitoring (HBPM) was used to measure BP in triplicate in the morning and evening using an OMRON M3 device for 5 days within a 10-day period, and the average of readings from the last 4 days was used to establish eligibility for the trial. The trial enrolled participants with hypertension, without diabetes, eGFR >60 mL/min/1.73 m^2^, aged ≥18 years with 4 days of mean HBPM systolic blood pressure (SBP) >135 mm Hg and HBPM diastolic blood pressure (DBP) >85 mm Hg despite treatment with at least 1 nondiuretic antihypertensive drug for at least 3 months before enrollment. Treatment was established from participant’s reported medication history and GP prescription information. A salivary sample was obtained for genotyping the rs13333226 SNP with the investigators and trial participants masked to the participant’s genotype. The consent process was in 2 steps. Initial consent for screening was completed electronically with written informed consent given by participants at the initial study visit. The protocol was approved by the West of Scotland Research Ethics Committee 5 (16/WS/0160). There was no data-monitoring board. The trial is registered with ClinicalTrials.gov number NCT03354897. There was disruption to the study during the COVID-19 pandemic, which led to the disruption of some of the follow-up visits for participants and an extension of the overall study time to allow for the completion of the protocol.

### Procedures

Participants were confirmed to have uncontrolled hypertension while on therapy with 1 or more antihypertensive drugs for at least 3 months. They should not have had any changes in antihypertensive treatment or dosages in the preceding 3 months. If a participant was on diuretic therapy (loop, thiazide, or thiazide-like diuretics), they underwent a washout period of 2 weeks before the baseline visit. All participants were prescribed torasemide (5 mg) daily for 16 weeks. Treatment interruptions of ≥21 days resulted in withdrawal from the study, while interruption, of <21 days led to a commensurate lengthening of the current study period. After initial screening and enrollment, there were 3 subsequent visits on medication at 2, 8, and 16 weeks. Ambulatory BP monitoring (ABPM) over 24 hours using Spacelabs 90217RM was measured at baseline and then at the end of the 2 subsequent periods of study (weeks 8 and 16). ABPM measured daytime readings every 30 minutes (08:00–21:59) and nighttime readings every 60 minutes (22:00–07:59). ABPM was deemed valid if ≥14 daytime measurements were obtained. With the exception of the baseline ABPM, all participants must have been taking the study drug for at least 28 days before ABPM. During the study, we applied stringent criteria to assess the quality of ABPM recordings to ensure good quality ABPM data was obtained. If there was any evidence of nonfulfillment of these criteria, this resulted in repeated ABPM measurements. All ABPM measurements that were included in the analyses met these quality standards. Our justification to reduce the nighttime frequency of ABPM measurements was to ensure greater compliance and completion of the full 24-hour ABPM by all study participants. HBPM was measured at weeks 4 and 12 to provide additional data points between ABPM measurements at baseline, week 8, and week 16. This approach aimed to balance the need for comprehensive BP data across the follow-up period with minimal patient discomfort. HBPM measurements were taken over a 7-day period and averaged, providing a reliable surrogate for ABPM, particularly for daytime readings. Office BP measurements were collected at all study visits (baseline, weeks 8 and 16).

### Outcomes

The primary outcome was the difference in overall mean 24-hour ABPM SBP. Secondary outcomes were 24-hour ABPM DBP, daytime ABPM SBP and DBP, nighttime ABPM SBP and DBP, HBPM SBP and DBP, and serum electrolytes. In our protocol, we planned to compare changes in outcomes over time between groups using baseline-adjusted regression models. However, given that the groups being compared are not randomized, an alternative method was specified in the final Statistical Analysis Plan before conducting the analysis (Supplemental Material). Our primary analysis was modified to account for potential differences in baseline SBP, as there could have been differences in this study. Unlike a randomized controlled trial, where any observed difference is assumed to be random, in this study, we anticipated a potential difference based on previous GWAS studies. Our inclusion criteria, which required a high SBP at baseline, resulted in a truncated distribution at the lower end, attenuating but not entirely eliminating the expected difference.

### Statistical Analysis

Sample size calculations were based on standard formulae for a normally distributed outcome, for detecting a difference in SBP between groups, assuming a SD of 8 mm Hg, a ratio of AA to AG/GG participants of 2:1 based on expected allele frequencies in the study source population, and a successful follow-up of 80% of recruited participants. Under these assumptions, recruitment of 240 participants would have 90% power to detect a 4 mm Hg SBP difference and 81% power to detect a 3.5 mm Hg difference between groups for the primary outcome.^16^

Continuous data are summarized as mean and SD or median and inter-quartile range (defined as the first and third quartiles). Categorical data are summarized as frequencies and percentages.

We tested hypotheses using mixed-effects linear regression models, utilizing data from baseline and all follow-up time points at which relevant data were available. All models included a random effect for each subject. Analyses of SBP trajectories are detailed in Supplemental Methods.

Analyses were conducted in the per-protocol dataset, defined as those participants who appeared to be adherent with treatment based on having taken at least 80% of allocated medication. The primary analysis of 24-hour mean ABPM SBP was repeated in the full analysis set of all participants as a sensitivity analysis.

We performed additional post hoc analyses modeling all available daytime SBP measurements (from ABPM, HBPM, and office BP measurements) recorded at or after baseline as a function of time since baseline. The model included a random effect for the subject and fixed effects for genotype (AA versus AG/GG), type of measurement (ABPM, HBPM, or office BP), age, sex, and body mass index. The effect of time since baseline was modeled using a cubic spline to capture potential nonlinear trends.

### Role of the Funding Source

The funders had no role in the data collection, data analysis, data interpretation, or the writing of the report. The investigators and all authors had sole discretion in the data analysis and interpretation, the writing of the report, and the decision to submit it for publication. The corresponding author had full access to all the data and the final responsibility to submit it for publication.

## RESULTS

Between May 25, 2017, and May 24, 2021, we screened 251 participants, of whom 222 entered the study and were prescribed torasemide. After excluding 48 participants (no follow-up data: 39; missing data or <80% adherence: 5; missing genotype: 4), 174 participants who had genotype data and took at least 80% of the dispensed torasemide over the study period were included in the per-protocol analysis. Table 1 shows the baseline characteristics of the per-protocol population, and the summary of the full analysis set of participants is presented in Table S1. The study participants were middle-aged (59±11 years), predominantly male (62%), obese (body mass index, 32±7 kg/m^2^), with normal eGFR (92±17 mL/min/1.73 m²) and an average baseline ABPM of 138/81 mm Hg.

**Table 1.**
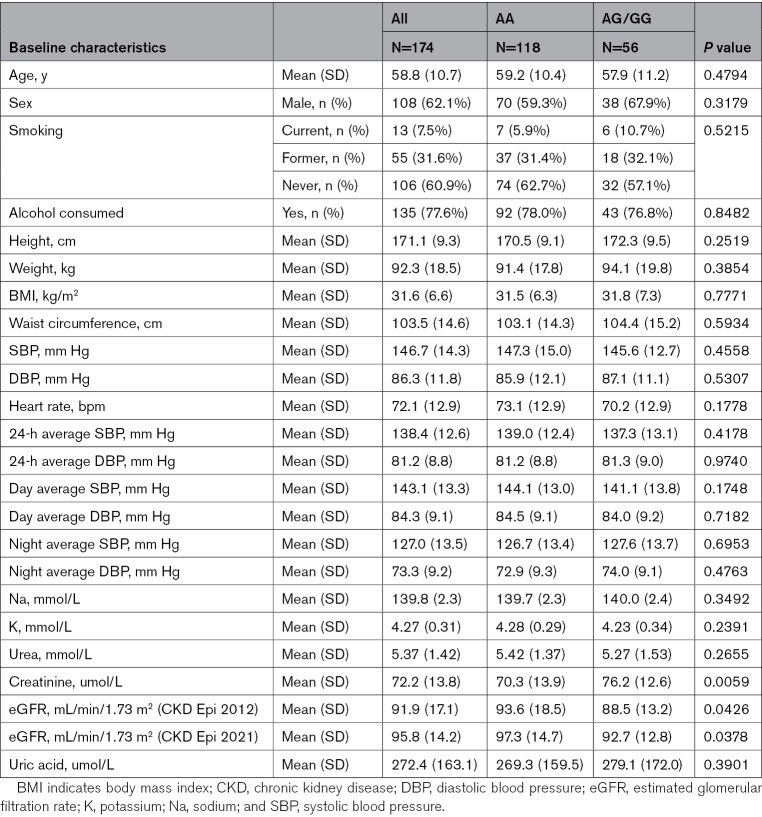
Baseline Characteristics of Study Population

### Primary Outcome

ABPM measures over time by genotype are summarized in Table 2 and the Figure. The mean baseline SBP was different between the AA and AG/GG groups (139.0±12.4 versus 137.3±13.1 mm Hg), but this was not statistically significant. Significant reductions in 24-hour mean ABPM SBP were observed in both groups after 16 weeks (AA, −6.57 mm Hg [95% CI, −8.44 to −4.69]; *P*<0.0001; AG/GG, −3.22 [95% CI, −5.93 to −0.51]; *P*=0.0209). The change in mean ABPM SBP from baseline to 16 weeks demonstrated that the AA genotype had a significantly greater response to torasemide compared with the AG/GG genotypes, with a difference of −3.35 ([95% CI, −6.64 to −0.05] mm Hg; *P*=0.0481; Table 2; Figure A and B). The patterns of SBP change over the study period varied by genotype (*P*=0.0035 for difference in trajectories; Table 3) with Figure B and C depicting, respectively, the mean predicted change from baseline of 24-hour ABPM SBP and ABPM daytime SBP. Model 3 showed the best model fit (lowest Akaike information criterion), suggesting that participants with the AG/GG genotype and participants with the AA genotype have different trajectories of 24-hour mean ABPM SBP over 16 weeks after initiating treatment with torasemide. The AA group showed a consistent, continuous decrease over the whole study period (from baseline to week 8: −4.34 [95% CI, −6.32 to −2.36]; *P*<0.0001; from weeks 8 to 16: −2.22 [95% CI, −4.24 to −0.21]; *P*=0.0320), whereas the AG/GG group displayed a more marked decrease at 8 weeks (−7.03 [95% CI, −9.85 to −4.19]; *P*<0.0001), followed by a rebound between 8 and 16 weeks (+3.81 [95% CI, 0.92 to 6.69]; *P*=0.0104).

**Table 2.**
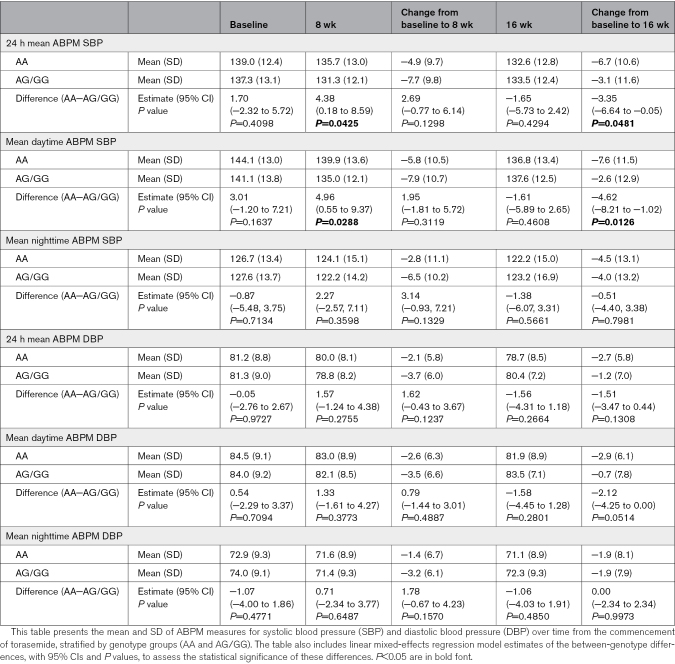
Ambulatory Blood Pressure Monitoring (ABPM) Measures Over Time by Genotype and Between-Genotype Differences

**Table 3.**
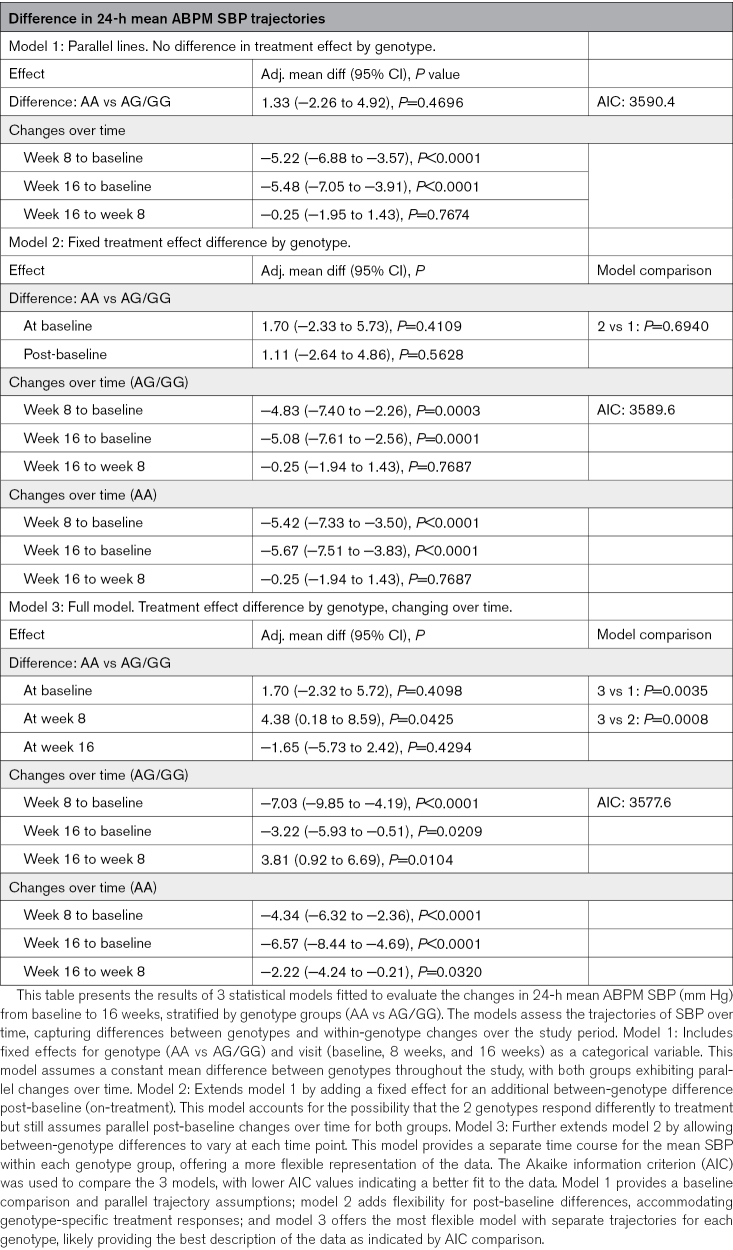
Changes in 24-Hour Mean Ambulatory Blood Pressure Monitoring (ABPM) Systolic Blood Pressure (SBP) Trajectories from Baseline to 16 Weeks by Genotype

**Figure.**
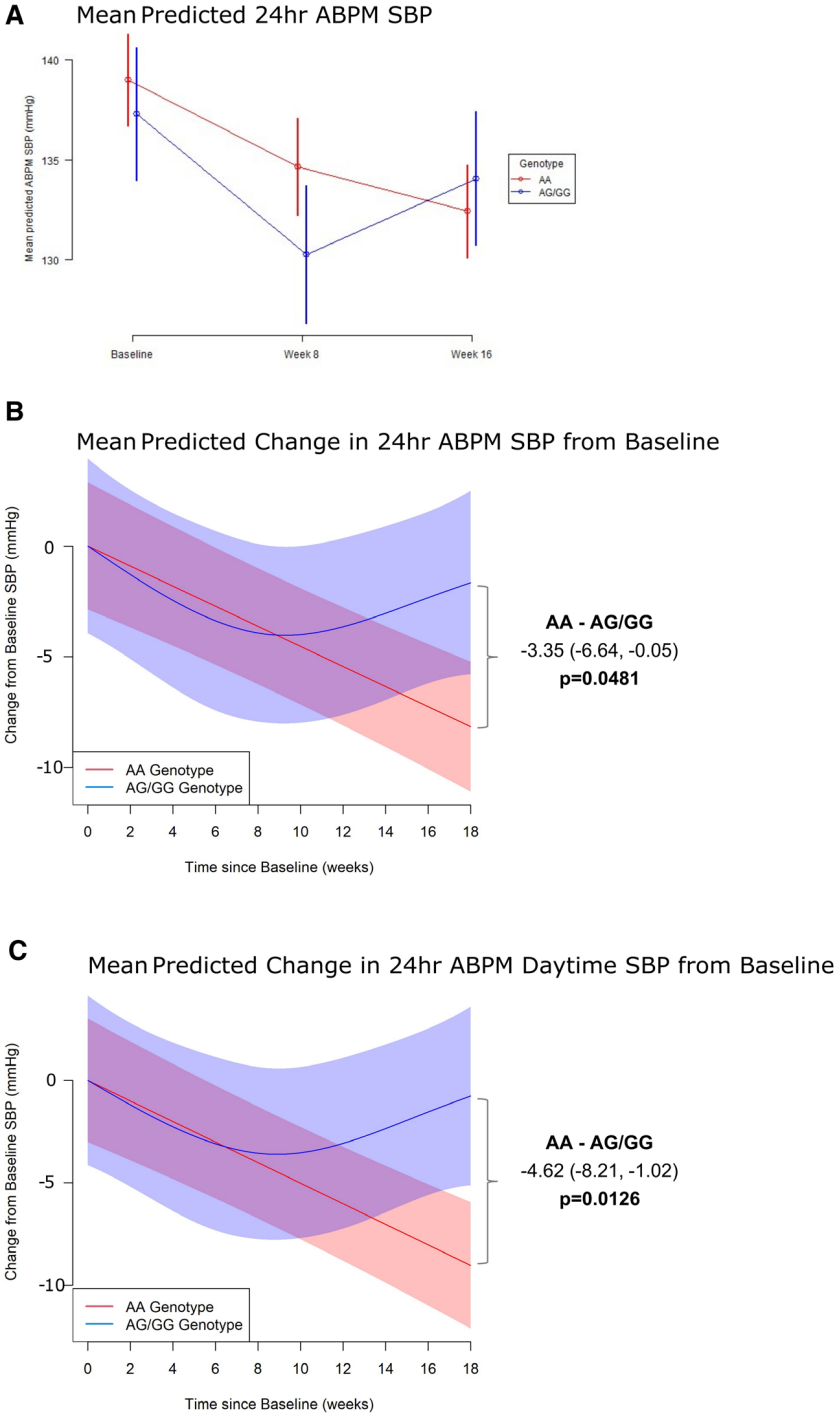
**Changes in 24-hour mean systolic blood pressure by genotype over time post-torasemide commencement.** This figure illustrates the variations in 24-hour mean systolic blood pressure (ABPM SBP) across different genotypes at 3 time points following torasemide commencement: baseline, 8 weeks, and 16 weeks. **A**, Displays the mean predicted ABPM SBP values for 2 genotype groups at baseline, 8 weeks, and 16 weeks. Each point represents the mean SBP, with error bars indicating the 95% CIs, providing a visual representation of the range within which the true mean SBP is expected to lie for each genotype group at each time point. **B**, Shows the change in mean predicted ABPM SBP from baseline for the 2 genotype groups at 8 weeks and 16 weeks. This panel highlights the difference in SBP from the initial baseline measurement, with 95% CIs to indicate the precision of the estimated changes over time for each genotype group. **C**, Presents the change in mean predicted daytime SBP from baseline for the 2 genotype groups at 8 weeks and 16 weeks. Similar to (**B**), this panel focuses on daytime SBP, providing insights into how daytime blood pressure changes relative to the baseline measurement over the study period, with 95% CIs to show the variability and reliability of the estimates.

Due to a significant difference in baseline eGFR between the AA and AG/GG groups, we conducted additional analyses adjusting for baseline eGFR. The results from these adjusted analyses show that incorporating baseline eGFR into the final model has virtually no impact on the model fit or the overall conclusions of our study (Table 4).

**Table 4.**
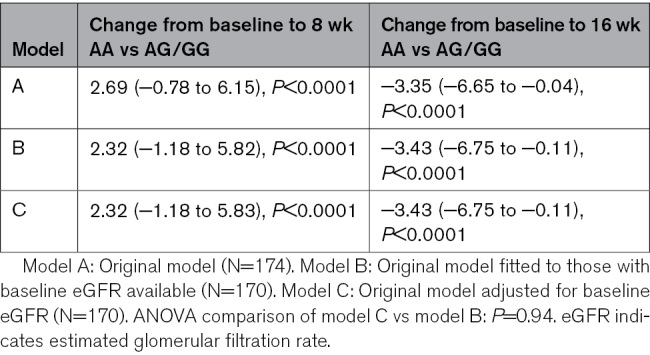
Comparison of Linear Mixed Model Estimates Adjusted for Baseline eGFR of the Differences Between Genotype Groups in the Change from Baseline ABPM SBP at 8 and 16 Weeks

Sensitivity analysis in the full analysis set including 131 AA and 65 AG/GG participants showed similar results (Table S1). Analysis by 3 genotypes (AA 118, AG 50, and GG 6) also showed similar results (Table S2).

### Post Hoc Analyses Combining Daytime ABPM, Home, and Office SBP Measurements

Figure S1 presents the results of post hoc analyses integrating all available BP data from ABPM (daytime), home, and office measurements. When considering all daytime SBP measurements, both the AA and AG/GG genotype groups exhibit an initial decline followed by a rebound (panels A, B, and C). The AA group declines more rapidly, reaching its lowest point just after 4 weeks, with some rebound by the 8-week visit. The AG/GG group reaches its minimum later at around 8 weeks, showing a clear fall-rebound pattern. The daytime ABPM SBP plot in this analysis mirrors the 24-hour ABPM patterns reported in the primary analysis with the AA genotype group demonstrating a linear decline, whereas the AG/GG group shows a decline at 8 weeks followed by an increase. Examining the difference in SBP between the genotype groups (panel D), the AA genotype group appears to have a higher SBP at baseline. This difference decreases as the AA group experiences a more rapid decline for up to 4 weeks. The difference then increases as the AA group shows a rebound while the AG/GG group continues to decline. Beyond 8 weeks, the difference reduces again as the AA group plateaus and the AG/GG group rebounds, resulting in no significant difference between the groups after 16 weeks. Analyzing the difference in changes from baseline (panels E and F), the AA group shows larger predicted reductions at all time points, with a variable pattern: growing in the first month, shrinking in the second month, and increasing for the remainder of the study, leading to a statistically greater reduction in daytime SBP after 16 weeks.

### Secondary Outcomes

The changes in ABPM DBP over 16 weeks paralleled those seen in SBP, with AA genotype carriers experiencing a consistent reduction from 81.2±8.8 to 78.7±8.5 mm Hg. Conversely, the AG/GG genotype group’s DBP moved from 81.3±9 to 80.4±7.2 mm Hg at the study’s end, including an initial decrease at 8 weeks (−3.40 mm Hg [95% CI, −5.08 to −1.72]; *P*=0.0001) and a subsequent rise from 8 to 16 weeks (2.24 mm Hg [95% CI, 0.53–3.95]; *P*=0.0110). The difference in trajectories was statistically significant (*P*=0.0137), as shown in Table S3. Daytime mean ABPM SBP followed a similar pattern (Tables S4 and S5). However, no genotype-based differences were observed in nighttime mean SBP and DBP, as well as in home SBP and DBP measurements (Tables S6 through S9). There were no significant differences by genotype for eGFR, serum sodium, serum potassium, serum creatinine, and serum uric acid levels (Tables S10 through S16).

### Adverse Events

Torasemide was well tolerated with similarly low rates of adverse events and withdrawals due to adverse events (Table S17 through S21 show the numbers and rates of the commonest adverse events and withdrawal reasons). Three participants had serious adverse events, which were thought to be related to the treatment, in the different genotype groups (2 in the AA group and 1 in the AG/GG group): back pain due to degenerative joint disease; requiring hospitalization; non-ST-elevation myocardial infarction; and acute kidney injury.

## DISCUSSION

This multicenter trial provides evidence of a genotype-specific response to torasemide in participants with hypertension, highlighting the role of *UMOD* genetic variation in modulating the efficacy of loop diuretics through the UMOD-NKCC2 pathway. Participants with the rs13333226-AA genotype exhibited a consistent reduction in SBP over 16 weeks compared with those with the AG/GG genotype, who experienced an initial decrease followed by a rebound. These findings suggest the potential for incorporating *UMOD* genotype information into personalized hypertension treatment strategies, aiming to optimize the use of loop diuretic therapy.

The allele frequencies of the A and G alleles in our trial population are 81.3% and 18.6%, respectively. These frequencies closely mirror those observed in large cohorts, such as 1000Genomes-European (A: 80.8%; G: 19.2%), TwinsUK (A: 80.6%; G: 19.3%), and ALSPAC (A: 80.9%; G: 19.0%) (https://genome.ucsc.edu/). Importantly, there were no significant differences in allele frequencies between our trial cohort and the published cohorts (*P*=0.46). In our cohort of uncontrolled hypertensive patients, we observed that baseline SBP differed between the 2 genotype groups. Specifically, SBP was lower in the G allele carriers compared with the AA group, though this difference was not statistically significant. This finding aligns with larger population cohort GWAS studies. Our inclusion criteria required selecting hypertensive patients with SBP above a certain threshold, resulting in a truncated distribution at the lower end. This truncation attenuated but did not entirely eliminate the expected difference in SBP between the genotype groups.

Trudu et als^12^ earlier retrospective study found that individuals with hypertension who were homozygous for the A allele showed a more pronounced response to furosemide at 4 hours posttreatment than those with the alternative genotypes. Building upon these initial findings, our study corroborates the enhanced efficacy of loop diuretics in participants homozygous for the *UMOD*-increasing allele. The initial decrease in BP followed by a rebound observed in G allele carriers hints at a heightened sensitivity to torasemide, possibly suggesting an underlying activation of the renin-angiotensin-aldosterone system (RAAS). The absence of direct renin and aldosterone measurements, coupled with the lack of stored samples, renders our hypothesis on RAAS activation speculative, marking a limitation of our study. Nonetheless, existing literature supports the merit of further investigation, particularly in light of research linking loop diuretic resistance in heart failure to RAAS and sympathetic nervous system activation.^17,18^ Additionally, findings from mouse models showing uromodulin overexpression leads to reduced renin and cyclooxygenase-2 levels suggest a potential parallel in humans that could explain the genotype-dependent BP responses observed.^6^ The *UMOD* genotype has also been associated with renal function, and Mendelian randomization studies show higher urinary uromodulin levels are causally associated with lower eGFR and higher BP, with only 28% of the uromodulin-BP effect mediated through eGFR.^10^ While there were no significant genotype-related differences in kidney function in this trial, a lower baseline eGFR observed in the AG/GG group requires further exploration. This could reflect a lower BP or be a chance finding due to the limited sample size.

Our additional post hoc analyses modeling all available daytime SBP measurements (from ABPM, HBPM, and office BP measurements) confirm that the AG/GG group exhibits a fall-rebound pattern similar to the primary 24-hour ABPM analysis. The robustness of our findings is supported by modeling all available daytime SBP data, mitigating concerns about missing observations, and ensuring the quality of ABPM data. The consistent patterns observed across ABPM, HBPM, and office BP measurements suggest that the lack of significant differences in HBPM may be due to the different measurement methodologies and intervals. However, the overall trends align with our primary findings. Thus, the pattern we observe may suggest a genetically driven counterregulatory effect, as hypothesized. The fall-rebound effect, which is prominent in the AG/GG group, warrants further investigations with measurements of renin and aldosterone levels along with urinary sodium excretion measurements in future studies.

Despite the availability of regularly updated international treatment guidelines for hypertension management, considerable clinical uncertainty persists about the optimal management of individuals with uncontrolled or treatment-resistant hypertension. Diuretics have long been established as cornerstone agents in the management of hypertension, with robust evidence supporting their efficacy in reducing BP and lowering cardiovascular risk. Thiazide/thiazide-like diuretics have been extensively studied and are recommended as first-line therapy in various international guidelines due to their effectiveness, low cost, and favorable outcomes in large-scale clinical trials. While there is consensus that resistant hypertension stems from excessive sodium retention and further diuretic therapy may be effective, the superiority of 1 diuretic type over another remains unstudied, with existing evidence limited to diuretics targeting the distal nephron (spironolactone). Loop diuretics, such as furosemide and torasemide, are commonly used in the management of fluid overload conditions like heart failure, renal failure, and cirrhosis but are less frequently prescribed for hypertension due to concerns about electrolyte disturbances and adverse metabolic effects.

Given that volume overload is a primary contributor to resistant hypertension, directing loop diuretics to individuals more likely to respond could offer a more targeted approach for uncontrolled hypertension management. Establishing that hypertensive patients with uncontrolled hypertension harboring the genetic variant associated with increased uromodulin production—present in ≈67% of the population—exhibit enhanced responsiveness to loop diuretics could potentially advance clinical practice. Our findings indicate that individuals with uncontrolled hypertension carrying the rs13333226-AA genotype experience more consistent reductions in SBP over a 16-week period compared with those carrying the G allele. This suggests a potential therapeutic advantage of loop diuretics in hypertensive patients homozygous for the *UMOD*-increasing allele.

A limitation of our trial protocol is the absence of evaluations for uromodulin levels and NKCC2 activity, which restricts our ability to fully elucidate the observed relationship between torasemide response and *UMOD* genotype. Despite this limitation, our study provides important evidence supporting a plausible interaction between NKCC2 and uromodulin, which should catalyze future mechanistic studies. Other limitations include excluding participants with diabetes and lower eGFR and studying a predominantly White Caucasian population. Patients with an eGFR below 60 mL/min/1.73 m² were excluded from the trial for multiple reasons. Firstly, the accurate interpretation of the BP response to loop diuretics may be compromised in individuals with diminished renal function, given the underlying renal pathology affecting renal hemodynamics and fluid balance. Furthermore, the underlying renal pathology may contribute to uncontrolled hypertension independently of the efficacy of antihypertensive medications. Additionally, loop diuretics are commonly indicated for volume management in patients with reduced eGFR, often necessitating higher doses to achieve therapeutic efficacy. Moreover, the *UMOD* genotype has been associated with eGFR and the development of CKD. The exclusion of patients with diabetes from the trial is justified by several considerations. The inclusion of diabetic individuals could introduce a confounding variable, particularly given the stringent BP targets often required to mitigate the risk of diabetic complications. Additionally, uncontrolled hypertension in diabetes may signify underlying secondary etiologies such as renal artery stenosis, requiring different management strategies. By excluding patients with diabetes and those with eGFR <60 mL/min/1.73 m², the trial aims to establish a more homogeneous study population, thereby minimizing potential confounding factors and enhancing the validity of the trial findings in attributing any observed BP responses to the interaction between *UMOD* genotype and loop diuretic therapy.

Future studies should investigate the generalizability of these findings to diverse groups and include morbidity and mortality outcomes. Additionally, measuring plasma renin and aldosterone could clarify the potential role of RAAS activation in BP trajectories. While we utilized ABPM to quantify the diuretic response in this study, future research could benefit from assessing sodium excretion rate as a function of tubular delivery of diuretic by genotype, providing a valuable metric for assessing diuretic efficacy. Including a randomized group with nonloop diuretics in future studies could help compare the efficacy of different diuretics used in hypertension treatment.

Despite limitations, our study has several strengths, including the use of HBPM to confirm uncontrolled hypertension during screening and ABPM for precise outcome assessment. The genotype blinding and 2-step consent process also strengthened the study design, improving efficiency and reducing screen failures.

### Conclusions

This study enhances our understanding of the UMOD-NKCC2 interaction and its impact on loop diuretic response and BP. Our findings underscore the potential for genotype-guided use of loop diuretics in hypertension management. This should instigate pragmatic trials to explore the earlier use of torasemide as a third-line agent in AA genotype patients with resistant hypertension. Further research is needed to investigate the underlying mechanisms, particularly the role of RAAS activation in G allele carriers.

### Perspectives

This study represents the first clinical trial to test a GWAS SNP identified from genome-wide association studies of BP and hypertension, among the over 2000 GWAS SNPs discovered for BP to date. Our findings advance the understanding of uromodulin’s role in BP regulation and emphasize the genotype-specific interaction between UMOD and NKCC2. These insights suggest the potential for personalizing hypertension therapy based on genetic information, leading to more effective and tailored treatment strategies. Future research should continue to explore the clinical and physiological implications of these genetic interactions, as well as investigate other GWAS signals to identify new pathways that can be translated into clinical practice to improve patient outcomes in hypertension.

## ARTICLE INFORMATION

### Acknowledgments

The authors would like to thank all the participants from all sites who took part in this study. The authors also would like to acknowledge the efforts of our clinical research trial nurses who helped with the study in the respective sites—Glasgow: Hayley King, Lesley Gilmour, Siouxsie Mackenzie, Kirsty Fallon, Ammani Brown; Edinburgh: Vanessa Melville; Dundee: Caroline Hall, Wendy Urquhart.

### Author Contributions

S. Padmanabhan conceived and designed the project. S. Padmanabhan and L. McCallum wrote the protocol and obtained ethical approval. A. McConnachie analyzed the data. All authors interpreted the data. L. McCallum and S. Lip wrote the first draft of the manuscript. All authors provided critical revisions to the final manuscript for submission.

### Sources of Funding

This study was supported by British Heart Foundation (CS/16/1/31878, S. Padmanabhan, D.J. Webb, T.M. MacDinald, A. McConnachie). L. McCallum is supported by NHS Research Scotland Career Researcher Fellowship. S. Lip is supported by Heart Research UK (RG2690/21/24). S. Padmanabhan is supported by the British Heart Foundation Centre of Excellence Award (RE/18/6/34217) and the United Kingdom Research and Innovation Strength in Places Fund (SIPF00007/1).

### Disclosures

I.S. Mackenzie is a member of the British Heart Foundation Clinical Studies Committee. The other authors report no conflicts.

### Supplemental Material

Supplemental Methods

Tables S1–S21

Figure S1

## Supplementary Material

## References

[1] Ettehad D Emdin CA Kiran A Anderson SG Callender T Emberson J Chalmers J Rodgers A Rahimi K. Blood pressure lowering for prevention of cardiovascular disease and death: a systematic review and meta-analysis. Lancet. 2016;387:957–967. doi: 10.1016/S0140-6736(15)01225-8 26724178 10.1016/S0140-6736(15)01225-8

[2] Forouzanfar MH Liu P Roth GA Ng M Biryukov S Marczak L Alexander L Estep K Hassen Abate K Akinyemiju TF. Global burden of hypertension and systolic blood pressure of at least 110 to 115 mm Hg, 1990-2015. JAMA. 2017;317:165–182. doi: 10.1001/jama.2016.19043 28097354 10.1001/jama.2016.19043

[3] Rethy L Shah NS Paparello JJ Lloyd-Jones DM Khan SS. Trends in hypertension-related cardiovascular mortality in the United States, 2000 to 2018. Hypertension. 2020;76:e23–e25. doi: 10.1161/HYPERTENSIONAHA.120.15153 32654559 10.1161/HYPERTENSIONAHA.120.15153 PMC9390965

[4] Iniesta R Campbell D Venturini C Faconti L Singh S Irvin MR Cooper-DeHoff RM Johnson JA Turner ST Arnett DK. Gene variants at loci related to blood pressure account for variation in response to antihypertensive drugs between black and white individuals. Hypertension. 2019;74:614–622. doi: 10.1161/HYPERTENSIONAHA.118.12177 31327267 10.1161/HYPERTENSIONAHA.118.12177

[5] Padmanabhan S Joe B. Towards precision medicine for hypertension: a review of genomic, epigenomic, and microbiomic effects on blood pressure in experimental rat models and humans. Physiol Rev. 2017;97:1469–1528. doi: 10.1152/physrev.00035.2016 28931564 10.1152/physrev.00035.2016 PMC6347103

[6] Bachmann S Mutig K Bates J Welker P Geist B Gross V Luft FC Alenina N Bader M Thiele BJ. Renal effects of Tamm-Horsfall protein (uromodulin) deficiency in mice. Am J Physiol Renal Physiol. 2005;288:F559–F567. doi: 10.1152/ajprenal.00143.2004 15522986 10.1152/ajprenal.00143.2004

[7] Graham LA Padmanabhan S Fraser NJ Kumar S Bates JM Raffi HS Welsh P Beattie W Hao S Leh S. Validation of uromodulin as a candidate gene for human essential hypertension. Hypertension. 2014;63:551–558. doi: 10.1161/HYPERTENSIONAHA.113.01423 24324041 10.1161/HYPERTENSIONAHA.113.01423

[8] Olden M Corre T Hayward C Toniolo D Ulivi S Gasparini P Pistis G Hwang S-J Bergmann S Campbell H. Common variants in UMOD associate with urinary uromodulin levels: a meta-analysis. J Am Soc Nephrol. 2014;25:1869–1882. doi: 10.1681/ASN.2013070781 24578125 10.1681/ASN.2013070781 PMC4116060

[9] Padmanabhan S Melander O Johnson T Di Blasio AM Lee WK Gentilini D Hastie CE Menni C Monti MC Delles C; Global BPgen Consortium. Genome-wide association study of blood pressure extremes identifies variant near UMOD associated with hypertension. PLoS Genet. 2010;6:e1001177. doi: 10.1371/journal.pgen.1001177 21082022 10.1371/journal.pgen.1001177 PMC2965757

[10] Ponte B Sadler MC Olinger E Vollenweider P Bochud M Padmanabhan S Hayward C Kutalik Z Devuyst O. Mendelian randomization to assess causality between uromodulin, blood pressure and chronic kidney disease. Kidney Int. 2021;100:1282–1291. doi: 10.1016/j.kint.2021.08.032 34634361 10.1016/j.kint.2021.08.032

[11] Pruijm M Ponte B Ackermann D Paccaud F Guessous I Ehret G Pechère-Bertschi A Vogt B Mohaupt MG Martin PY. Associations of urinary uromodulin with clinical characteristics and markers of tubular function in the general population. Clin J Am Soc Nephrol. 2016;11:70–80. doi: 10.2215/CJN.04230415 26683888 10.2215/CJN.04230415 PMC4702229

[12] Trudu M Janas S Lanzani C Debaix H Schaeffer C Ikehata M Citterio L Demaretz S Trevisani F Ristagno G; SKIPOGH Team. Common noncoding UMOD gene variants induce salt-sensitive hypertension and kidney damage by increasing uromodulin expression. Nat Med. 2013;19:1655–1660. doi: 10.1038/nm.3384 24185693 10.1038/nm.3384 PMC3856354

[13] Evangelou E Warren HR Mosen-Ansorena D Mifsud B Pazoki R Gao H Ntritsos G Dimou N Cabrera CP Karaman I; Million Veteran Program. Genetic analysis of over 1 million people identifies 535 new loci associated with blood pressure traits. Nat Genet. 2018;50:1412–1425. doi: 10.1038/s41588-018-0205-x 30224653 10.1038/s41588-018-0205-x PMC6284793

[14] Giri A Hellwege JN Keaton JM Park J Qiu C Warren HR Torstenson ES Kovesdy CP Sun YV Wilson OD; Understanding Society Scientific Group. Trans-ethnic association study of blood pressure determinants in over 750,000 individuals. Nat Genet. 2019;51:51–62. doi: 10.1038/s41588-018-0303-9 30578418 10.1038/s41588-018-0303-9 PMC6365102

[15] Wuttke M Li Y Li M Sieber KB Feitosa MF Gorski M Tin A Wang L Chu AY Hoppmann A; Lifelines Cohort Study. A catalog of genetic loci associated with kidney function from analyses of a million individuals. Nat Genet. 2019;51:957–972. doi: 10.1038/s41588-019-0407-x 31152163 10.1038/s41588-019-0407-x PMC6698888

[16] McCallum L Brooksbank K McConnachie A Aman A Lip S Dawson J MacIntyre IM MacDonald TM Webb DJ Padmanabhan S. Rationale and design of the genotype-blinded trial of torasemide for the treatment of hypertension (BHF UMOD). Am J Hypertens. 2021;34:92–99. doi: 10.1093/ajh/hpaa166 33084880 10.1093/ajh/hpaa166 PMC7891239

[17] Ellison DH. Diuretic therapy and resistance in congestive heart failure. Cardiology. 2001;96:132–143. doi: 10.1159/000047397 11805380 10.1159/000047397

[18] Francis GS Siegel RM Goldsmith SR Olivari MT Levine TB Cohn JN. Acute vasoconstrictor response to intravenous furosemide in patients with chronic congestive heart failure. Activation of the neurohumoral axis. Ann Intern Med. 1985;103:1–6. doi: 10.7326/0003-4819-103-1-1 2860833 10.7326/0003-4819-103-1-1

