# Supplementary material for: UMOD Genotype-Blinded Trial of Ambulatory Blood Pressure Response to Torasemide
Source: Hypertension. 2024 Jul 30;81(10):2049–59. doi: 10.1161/HYPERTENSIONAHA.124.23122 (PMC11460757; doi:10.1161/HYPERTENSIONAHA.124.23122)

**Supplemental Material**

***UMOD* Genotype-Blinded Trial of Ambulatory Blood Pressure Response to Torasemide**

**Brief Title:** ***UMOD* and Torasemide Response: A Multicentre Trial**

**Authors**

Linsay McCallum^a,b^, Stefanie Lip^a,b^, Alex McConnachie^c^, Katriona Brooksbank^b^, Iain MacIntyre^d^, Alexander Doney^e^, Andrea Llano^a^, Alisha Aman^b^, Thomas M Caparrotta^d^, Gareth Ingram^a^, Isla S Mackenzie^e^, Anna F Dominiczak^b^, Thomas M MacDonald^e^, David J Webb^d^, Sandosh Padmanabhan^a,b^.

**Affiliations**

^a^Queen Elizabeth University Hospital, Glasgow, Scotland, United Kingdom

^b^School of Cardiovascular and Metabolic Health, University of Glasgow, Glasgow, Scotland, United Kingdom

^c^Robertson Centre for Biostatistics, School of Health and Wellbeing, University of Glasgow, Glasgow, United Kingdom.

^d^Clinical Pharmacology Unit and Research Centre, University of Edinburgh/BHF Centre of Research

Excellence, Edinburgh, United Kingdom.

^e^MEMO Research, University of Dundee, Ninewells Hospital and Medical School, Dundee, United Kingdom.

**Corresponding author**

Professor Sandosh Padmanabhan

Pontecorvo Chair of Pharmacogenomics

BHF Glasgow Cardiovascular Research Centre

School of Cardiovascular and Metabolic Health,

126 University Place

Glasgow

United Kingdom

G12 8TA

T: 0141 3302228

E: [Sandosh.Padmanabhan@glasgow.ac.uk](mailto:Sandosh.Padmanabhan@glasgow.ac.uk)

**Supplemental Methods**

**Analyses of SBP trajectories**

Three models were fitted for each outcome. Model 1: Includes fixed effects for genotype (AA vs. AG/GG) and visit (as a categorical variable). This model assumes a fixed mean difference between genotypes at baseline and throughout the study, with both groups varying over time in a parallel manner. Model 2: Same as Model 1, with a fixed effect to model an additional between-genotype difference post-baseline (i.e. on-treatment). This model accommodates for the possibility of a different response to treatment in the two genotypes, but otherwise the post-baseline changes over time are parallel. Model 3: Building on Model 2, the between-genotype differences are allowed to vary at all time points. Alternatively, this model can be seen as the mean outcome having a separate time course within each genotype group. Models 1, 2, and 3, were compared using the Akaike Information Criterion (AIC) to determine which model gives the best description of the data. Each model provides an estimate of the between-genotype difference in mean outcome at each time point, and the mean changes over time within each genotype, with 95% confidence intervals (CIs). Model 3 for each outcome was used to estimate the mean value of each outcome at each time point, with 95% CIs.

**Table S1**: Baseline characteristics of study population: Full Analysis Set.

|  |  | **All** | **AA** | **AG/GG** | **Missing** | **p-value** |
| --- | --- | --- | --- | --- | --- | --- |
|  |  | N = 222 | N = 131 | N = 65 | N = 26 |  |
| Age (years) | Mean (SD) | 58.8 (10.8) | 59.2 (10.4) | 57.9 (10.8) | 58.8 (13.1) | p=0.7423 |
| Gender | N (%) Male | 138 (62.7%) | 78 (59.5%) | 46 (70.8%) | 14 (58.3%) | p=0.2814 |
| Smoking | N (%) Current | 17 (7.7%) | 9 (6.9%) | 6 (9.2%) | 2 (7.7%) | p=0.9711 |
|  | N (%) Former | 70 (31.5%) | 41 (31.3%) | 21 (32.3%) | 8 (30.8%) |  |
|  | N (%) Never | 135 (60.8%) | 81 (61.8%) | 38 (58.5%) | 16 (61.5%) |  |
| Alcohol | N (%) Yes | 171 (77.0%) | 100 (76.3%) | 51 (78.5%) | 20 (76.9%) | p=0.9722 |
| Height (cm) | Mean (SD) | 170.9 (9.3) | 170.4 (9.1) | 172.7 (9.0) | 168.6 (10.4) | p=0.1124 |
| Weight (kg) | Mean (SD) | 92.1 (18.5) | 90.8 (18.0) | 94.4 (19.4) | 92.7 (19.0) | p=0.4503 |
| BMI (kg / m2) | Mean (SD) | 31.6 (6.4) | 31.3 (6.3) | 31.8 (7.0) | 32.5 (5.6) | p=0.6783 |
| Waist circumference (cm) | Mean (SD) | 103.5 (15.1) | 102.7 (15.1) | 104.1 (15.1) | 106.0 (15.6) | p=0.5454 |
| SBP (mmHg) | Mean (SD) | 145.3 (14.8) | 145.8 (15.4) | 144.8 (13.1) | 143.9 (16.6) | p=0.8143 |
| DBP (mmHg) | Mean (SD) | 85.5 (12.0) | 85.2 (12.2) | 86.4 (11.9) | 84.3 (11.9) | p=0.7239 |
| Heart rate (bpm) | Mean (SD) | 72.4 (13.0) | 73.2 (12.8) | 70.6 (13.7) | 72.3 (11.9) | p=0.4174 |
| 24h average SBP (mmHg) | Mean (SD) | 137.5 (12.5) | 138.6 (12.1) | 136.6 (13.2) | 134.0 (12.1) | p=0.1872 |
| 24h average DBP (mmHg) | Mean (SD) | 81.1 (9.0) | 81.1 (8.9) | 81.0 (9.3) | 81.2 (9.2) | p=0.9948 |
| Day average SBP (mmHg) | Mean (SD) | 142.0 (13.1) | 143.5 (12.9) | 140.2 (13.8) | 139.1 (11.4) | p=0.1227 |
| Day average DBP (mmHg) | Mean (SD) | 84.2 (9.5) | 84.3 (9.4) | 83.6 (9.6) | 85.6 (10.0) | p=0.6678 |
| Night average SBP (mmHg) | Mean (SD) | 126.1 (14.0) | 126.4 (12.9) | 127.5 (14.2) | 121.5 (17.5) | p=0.1809 |
| Night average DBP (mmHg) | Mean (SD) | 73.0 (9.4) | 72.8 (9.3) | 74.1 (9.4) | 71.2 (10.1) | p=0.3953 |
| Na (mmol/L) | Mean (SD) | 139.8 (2.3) | 139.7 (2.3) | 140.0 (2.3) | 139.4 (1.9) | p=0.5391 |
| K (mmol/L) | Mean (SD) | 4.25 (0.34) | 4.26 (0.31) | 4.23 (0.35) | 4.24 (0.42) | p=0.6621 |
| Urea (mmol/L) | Mean (SD) | 5.35 (1.43) | 5.37 (1.37) | 5.34 (1.48) | 5.26 (1.64) | p=0.7159 |
| Creatinine (umol/L) | Mean (SD) | 72.0 (13.8) | 69.8 (13.9) | 77.0 (12.9) | 70.5 (13.3) | p=0.0022 |
| eGFR (mL/min/1.73m^2^; CKD Epi 2012) | Mean (SD) | 92.1 (16.5) | 93.9 (18.0) | 88.2 (13.0) | 93.0 (14.8) | p=0.0746 |
| eGFR (mL/min/1.73m^2^; CKD Epi 2021) | Mean (SD) | 96.0 (14.0) | 97.6 (14.4) | 92.4 (12.7) | 96.9 (14.0) | p=0.0469 |
| Uric acid (umol/L) | Mean (SD) | 286.4 (159.3) | 271.6 (157.6) | 291.3 (174.8) | 354.1 (105.8) | p=0.0574 |

**Table S2:** Primary outcome, sensitivity analysis: 24-hr mean ABPM SBP (mmHg) in Full Analysis Set

|  |  | All | AA | AG/GG | Missing |
| --- | --- | --- | --- | --- | --- |
| Baseline | Nobs (Nmiss)  Mean (SD)  Median (Q1, Q3)  [Min, Max] | 221 (1)  137.5 (12.5)  135.0 (129.0, 146.0)  [106.0, 175.0] | 131 (0)  138.6 (12.1)  136.0 (131.0, 146.5)  [109.0, 175.0] | 65 (0)  136.6 (13.2)  136.0 (129.0, 143.0)  [106.0, 169.0] | 25 (1)  134.0 (12.1)  132.0 (126.0, 136.0)  [121.0, 169.0] |
| Week 8 visit | Nobs (Nmiss)  Mean (SD)  Median (Q1, Q3)  [Min, Max] | 145 (77)  134.2 (12.7)  135.0 (126.0, 141.0)  [106.0, 174.0] | 96 (35)  135.7 (12.9)  135.0 (127.0, 143.2)  [106.0, 174.0] | 47 (18)  131.3 (12.1)  132.0 (121.5, 139.0)  [110.0, 158.0] | 2 (24)  130.0 (2.8)  130.0 (129.0, 131.0)  [128.0, 132.0] |
| Change from baseline  to week 8 visit | Nobs (Nmiss)  Mean (SD)  Median (Q1, Q3)  [Min, Max] | 145 (77)  -5.7 (9.7)  -5.0 (-13.0, 1.0)  [-28.0, 21.0] | 96 (35)  -4.8 (9.7)  -4.0 (-11.2, 1.2)  [-28.0, 21.0] | 47 (18)  -7.7 (9.8)  -9.0 (-14.5, -1.5)  [-25.0, 15.0] | 2 (24)  -2.0 (2.8)  -2.0 (-3.0, -1.0)  [-4.0, 0.0] |
| Week 16 visit | Nobs (Nmiss)  Mean (SD)  Median (Q1, Q3)  [Min, Max] | 166 (56)  133.0 (12.6)  132.0 (125.0, 141.0)  [105.0, 164.0] | 112 (19)  132.5 (12.7)  132.0 (124.0, 140.2)  [106.0, 164.0] | 53 (12)  133.5 (12.4)  134.0 (125.0, 141.0)  [105.0, 160.0] | 1 (25)  149.0 (-)  149.0 (149.0, 149.0)  [149.0, 149.0] |
| Change from baseline  to week 16 visit | Nobs (Nmiss)  Mean (SD)  Median (Q1, Q3)  [Min, Max] | 166 (56)  -5.5 (11.0)  -5.0 (-11.8, 1.0)  [-40.0, 25.0] | 112 (19)  -6.7 (10.5)  -7.0 (-12.2, -0.8)  [-40.0, 17.0] | 53 (12)  -3.1 (11.6)  -2.0 (-10.0, 3.0)  [-28.0, 25.0] | 1 (25)  -6.0 (-)  -6.0 (-6.0, -6.0)  [-6.0, -6.0] |
| Model 1: parallel lines. No difference in treatment effect by genotype. | | | | | AIC: 3780.9 |
| Effect | | Adj. mean diff (95% CI), p | |  | |
| Difference: AA vs. AG/GG | | 1.64 (-1.72, 5.00), p=0.3406 | |  | |
| Changes over time:  Week 8 - Baseline  Week 16 - Baseline  Week 16 - Week 8 | | -5.00 (-6.63, -3.36), p<0.0001  -5.32 (-6.87, -3.78), p<0.0001  -0.32 (-2.00, 1.35), p=0.7070 | |  | |
| Model 2: fixed treatment effect difference by genotype. | | | | | AIC: 3780.1 |
| Effect | | Adj. mean diff (95% CI), p | | Model Comparison | |
| Difference: AA vs. AG/GG  At Baseline  Post-baseline | | 2.01 (-1.72, 5.74), p=0.2930  1.36 (-2.23, 4.94), p=0.4591 | | 2 vs. 1: p=0.6552 | |
| Changes over time (AG/GG):  Week 8 - Baseline  Week 16 - Baseline  Week 16 - Week 8 | | -4.56 (-7.09, -2.03), p=0.0005  -4.88 (-7.37, -2.40), p=0.0001  -0.32 (-2.00, 1.35), p=0.7089 | |  | |
| Changes over time (AA):  Week 8 - Baseline  Week 16 - Baseline  Week 16 - Week 8 | | -5.22 (-7.09, -3.33), p<0.0001  -5.54 (-7.34, -3.73), p<0.0001  -0.32 (-2.00, 1.35), p=0.7089 | |  | |
| Model 3: full model. Treatment effect difference by genotype, changing over time. | | | | | AIC: 3767.7 |
| Effect | | Adj. mean diff (95% CI), p | | Model Comparison | |
| Difference: AA vs. AG/GG  At Baseline  At Week 8  At Week 16 | | 2.01 (-1.71, 5.73), p=0.2919  4.68 (0.63, 8.73), p=0.0247  -1.43 (-5.35, 2.49), p=0.4768 | | 3 vs. 1: p=0.0029  3 vs. 2 p=0.0007 | |
| Changes over time (AG/GG):  Week 8 - Baseline  Week 16 - Baseline  Week 16 - Week 8 | | -6.80 (-9.59, -4.00), p<0.0001  -3.00 (-5.66, -0.33), p=0.0291  3.80 (0.92, 6.67), p=0.0102 | |  | |
| Changes over time (AA):  Week 8 - Baseline  Week 16 - Baseline  Week 16 - Week 8 | | -4.13 (-6.08, -2.18), p<0.0001  -6.43 (-8.28, -4.59), p<0.0001  -2.30 (-4.31, -0.30), p=0.0253 | |  | |

| **Table S3:**  Secondary outcome: 24-hour mean ABPM DBP (mmHg) | | | | |
| --- | --- | --- | --- | --- |
|  |  | All | AA | AG/GG |
| Baseline | Nobs (Nmiss)  Mean (SD)  Median (Q1, Q3)  [Min, Max] | 174 (0)  81.2 (8.8)  81.0 (74.2, 88.0)  [56.0, 100.0] | 118 (0)  81.2 (8.8)  81.0 (74.2, 87.8)  [63.0, 100.0] | 56 (0)  81.3 (9.0)  82.0 (75.5, 88.2)  [56.0, 99.0] |
| Week 8 visit | Nobs (Nmiss)  Mean (SD)  Median (Q1, Q3)  [Min, Max] | 142 (32)  79.6 (8.1)  79.5 (76.0, 84.8)  [53.0, 103.0] | 95 (23)  80.0 (8.1)  79.0 (75.5, 85.5)  [60.0, 103.0] | 47 (9)  78.8 (8.2)  80.0 (76.0, 84.0)  [53.0, 91.0] |
| Change from baseline  to week 8 visit | Nobs (Nmiss)  Mean (SD)  Median (Q1, Q3)  [Min, Max] | 142 (32)  -2.6 (5.9)  -2.0 (-6.8, 1.0)  [-19.0, 17.0] | 95 (23)  -2.1 (5.8)  -2.0 (-6.0, 2.0)  [-17.0, 17.0] | 47 (9)  -3.7 (6.0)  -3.0 (-8.0, 0.0)  [-19.0, 8.0] |
| Week 16 visit | Nobs (Nmiss)  Mean (SD)  Median (Q1, Q3)  [Min, Max] | 163 (11)  79.2 (8.1)  80.0 (74.0, 84.0)  [56.0, 99.0] | 110 (8)  78.7 (8.5)  79.0 (73.0, 84.0)  [56.0, 99.0] | 53 (3)  80.4 (7.2)  82.0 (77.0, 85.0)  [64.0, 96.0] |
| Change from baseline  to week 16 visit | Nobs (Nmiss)  Mean (SD)  Median (Q1, Q3)  [Min, Max] | 163 (11)  -2.2 (6.3)  -2.0 (-6.5, 2.0)  [-19.0, 15.0] | 110 (8)  -2.7 (5.8)  -2.5 (-7.0, 1.0)  [-19.0, 12.0] | 53 (3)  -1.2 (7.0)  -2.0 (-6.0, 4.0)  [-19.0, 15.0] |
| Model 1: parallel lines. No difference in treatment effect by genotype. | | | | AIC: 3144.8 |
| Effect | | Adj. mean diff (95% CI), p | |  |
| Difference: AA vs. AG/GG | | -0.10 (-2.60, 2.39), p=0.9365 | |  |
| Changes over time:  Week 8 - Baseline  Week 16 - Baseline  Week 16 - Week 8 | | -2.32 (-3.29, -1.34), p<0.0001  -2.18 (-3.11, -1.25), p<0.0001  0.14 (-0.86, 1.13), p=0.7902 | |  |
| Model 2: fixed treatment effect difference by genotype. | | | | AIC: 3145.2 |
| Effect | | Adj. mean diff (95% CI), p | | Model Comparison |
| Difference: AA vs. AG/GG  At Baseline  Post-baseline | | -0.05 (-2.77, 2.67), p=0.9727  -0.13 (-2.71, 2.44), p=0.9190 | | 2 vs. 1: p=0.9217 |
| Changes over time (AG/GG):  Week 8 - Baseline  Week 16 - Baseline  Week 16 - Week 8 | | -2.26 (-3.77, -0.74), p=0.0039  -2.12 (-3.61, -0.63), p=0.0057  0.14 (-0.86, 1.13), p=0.7906 | |  |
| Changes over time (AA):  Week 8 - Baseline  Week 16 - Baseline  Week 16 - Week 8 | | -2.34 (-3.47, -1.21), p=0.0001  -2.21 (-3.30, -1.12), p=0.0001  0.14 (-0.86, 1.13), p=0.7906 | |  |
| Model 3: full model. Treatment effect difference by genotype, changing over time. | | | | AIC: 3136.7 |
| Effect | | Adj. mean diff (95% CI), p | | Model Comparison |
| Difference: AA vs. AG/GG  At Baseline  At Week 8  At Week 16 | | -0.05 (-2.76, 2.67), p=0.9727  1.57 (-1.24, 4.38), p=0.2755  -1.56 (-4.31, 1.18), p=0.2664 | | 3 vs. 1: p=0.0137  3 vs. 2 p=0.0034 |
| Changes over time (AG/GG):  Week 8 - Baseline  Week 16 - Baseline  Week 16 - Week 8 | | -3.40 (-5.08, -1.72), p=0.0001  -1.16 (-2.76, 0.45), p=0.1596  2.24 (0.53, 3.95), p=0.0110 | |  |
| Changes over time (AA):  Week 8 - Baseline  Week 16 - Baseline  Week 16 - Week 8 | | -1.78 (-2.96, -0.60), p=0.0033  -2.67 (-3.79, -1.56), p<0.0001  -0.89 (-2.09, 0.30), p=0.1466 | |  |

| **Table S4:** Secondary outcome: 24-hour mean ABPM daytime SBP (mmHg) | | | | |
| --- | --- | --- | --- | --- |
|  |  | All | AA | AG/GG |
| Baseline | Nobs (Nmiss)  Mean (SD)  Median (Q1, Q3)  [Min, Max] | 173 (1)  143.1 (13.3)  141.0 (134.0, 150.0)  [106.0, 181.0] | 117 (1)  144.1 (13.0)  142.0 (137.0, 152.0)  [113.0, 181.0] | 56 (0)  141.1 (13.8)  140.0 (131.8, 146.2)  [106.0, 176.0] |
| Week 8 visit | Nobs (Nmiss)  Mean (SD)  Median (Q1, Q3)  [Min, Max] | 142 (32)  138.3 (13.3)  138.0 (129.0, 146.0)  [108.0, 187.0] | 95 (23)  139.9 (13.6)  140.0 (131.0, 148.0)  [108.0, 187.0] | 47 (9)  135.0 (12.1)  136.0 (124.0, 143.0)  [113.0, 164.0] |
| Change from baseline  to week 8 visit | Nobs (Nmiss)  Mean (SD)  Median (Q1, Q3)  [Min, Max] | 142 (32)  -6.5 (10.5)  -7.0 (-13.8, 0.0)  [-32.0, 23.0] | 95 (23)  -5.8 (10.5)  -5.0 (-12.0, 1.5)  [-32.0, 23.0] | 47 (9)  -7.9 (10.7)  -9.0 (-15.0, -1.0)  [-30.0, 18.0] |
| Week 16 visit | Nobs (Nmiss)  Mean (SD)  Median (Q1, Q3)  [Min, Max] | 163 (11)  137.1 (13.1)  136.0 (128.0, 146.0)  [107.0, 173.0] | 110 (8)  136.8 (13.4)  136.0 (128.0, 145.0)  [108.0, 173.0] | 53 (3)  137.6 (12.5)  137.0 (129.0, 148.0)  [107.0, 168.0] |
| Change from baseline  to week 16 visit | Nobs (Nmiss)  Mean (SD)  Median (Q1, Q3)  [Min, Max] | 163 (11)  -6.0 (12.1)  -7.0 (-12.0, 2.0)  [-43.0, 31.0] | 110 (8)  -7.6 (11.5)  -8.0 (-13.0, 0.8)  [-43.0, 26.0] | 53 (3)  -2.6 (12.9)  -1.0 (-11.0, 5.0)  [-28.0, 31.0] |
| Model 1: parallel lines. No difference in treatment effect by genotype. | | | | AIC: 3647.6 |
| Effect | | Adj. mean diff (95% CI), p | |  |
| Difference: AA vs. AG/GG | | 1.99 (-1.72, 5.70), p=0.2944 | |  |
| Changes over time:  Week 8 - Baseline  Week 16 - Baseline  Week 16 - Week 8 | | -5.86 (-7.67, -4.05), p<0.0001  -5.99 (-7.71, -4.27), p<0.0001  -0.12 (-1.97, 1.71), p=0.8951 | |  |
| Model 2: fixed treatment effect difference by genotype. | | | | AIC: 3645.9 |
| Effect | | Adj. mean diff (95% CI), p | | Model Comparison |
| Difference: AA vs. AG/GG  At Baseline  Post-baseline | | 3.01 (-1.21, 7.23), p=0.1647  1.40 (-2.50, 5.29), p=0.4831 | | 2 vs. 1: p=0.3237 |
| Changes over time (AG/GG):  Week 8 - Baseline  Week 16 - Baseline  Week 16 - Week 8 | | -4.79 (-7.59, -1.97), p=0.0010  -4.90 (-7.66, -2.15), p=0.0006  -0.12 (-1.96, 1.72), p=0.8994 | |  |
| Changes over time (AA):  Week 8 - Baseline  Week 16 - Baseline  Week 16 - Week 8 | | -6.39 (-8.48, -4.30), p<0.0001  -6.51 (-8.52, -4.50), p<0.0001  -0.12 (-1.96, 1.72), p=0.8994 | |  |
| Model 3: full model. Treatment effect difference by genotype, changing over time. | | | | AIC: 3633.7 |
| Effect | | Adj. mean diff (95% CI), p | | Model Comparison |
| Difference: AA vs. AG/GG  At Baseline  At Week 8  At Week 16 | | 3.01 (-1.20, 7.21), p=0.1637  4.96 (0.55, 9.37), p=0.0288  -1.61 (-5.89, 2.65), p=0.4608 | | 3 vs. 1: p=0.0024  3 vs. 2 p=0.0009 |
| Changes over time (AG/GG):  Week 8 - Baseline  Week 16 - Baseline  Week 16 - Week 8 | | -7.18 (-10.26, -4.09), p<0.0001  -2.87 (-5.83, 0.08), p=0.0588  4.31 (1.15, 7.45), p=0.0079 | |  |
| Changes over time (AA):  Week 8 - Baseline  Week 16 - Baseline  Week 16 - Week 8 | | -5.22 (-7.38, -3.06), p<0.0001  -7.49 (-9.54, -5.44), p<0.0001  -2.27 (-4.47, -0.07), p=0.0452 | |  |

| **Table S5:** Secondary outcome: 24- hour mean ABPM daytime DBP (mmHg) | | | | |
| --- | --- | --- | --- | --- |
|  |  | All | AA | AG/GG |
| Baseline | Nobs (Nmiss)  Mean (SD)  Median (Q1, Q3)  [Min, Max] | 173 (1)  84.3 (9.1)  84.0 (78.0, 91.0)  [57.0, 107.0] | 117 (1)  84.5 (9.1)  84.0 (78.0, 91.0)  [64.0, 107.0] | 56 (0)  84.0 (9.2)  84.0 (78.0, 91.2)  [57.0, 103.0] |
| Week 8 visit | Nobs (Nmiss)  Mean (SD)  Median (Q1, Q3)  [Min, Max] | 142 (32)  82.7 (8.7)  83.0 (77.2, 88.8)  [53.0, 105.0] | 95 (23)  83.0 (8.9)  82.0 (77.0, 89.5)  [64.0, 105.0] | 47 (9)  82.1 (8.5)  83.0 (79.0, 87.5)  [53.0, 95.0] |
| Change from baseline  to week 8 visit | Nobs (Nmiss)  Mean (SD)  Median (Q1, Q3)  [Min, Max] | 142 (32)  -2.9 (6.4)  -2.0 (-7.0, 1.0)  [-25.0, 18.0] | 95 (23)  -2.6 (6.3)  -2.0 (-6.0, 1.0)  [-17.0, 18.0] | 47 (9)  -3.5 (6.6)  -3.0 (-7.0, 0.0)  [-25.0, 10.0] |
| Week 16 visit | Nobs (Nmiss)  Mean (SD)  Median (Q1, Q3)  [Min, Max] | 163 (11)  82.4 (8.4)  83.0 (77.0, 88.5)  [58.0, 104.0] | 110 (8)  81.9 (8.9)  82.0 (76.0, 89.0)  [58.0, 104.0] | 53 (3)  83.5 (7.1)  84.0 (79.0, 88.0)  [66.0, 96.0] |
| Change from baseline  to week 16 visit | Nobs (Nmiss)  Mean (SD)  Median (Q1, Q3)  [Min, Max] | 163 (11)  -2.2 (6.8)  -2.0 (-7.0, 3.0)  [-18.0, 16.0] | 110 (8)  -2.9 (6.1)  -3.0 (-7.0, 1.0)  [-17.0, 12.0] | 53 (3)  -0.7 (7.8)  -1.0 (-6.0, 4.0)  [-18.0, 16.0] |
| Model 1: parallel lines. No difference in treatment effect by genotype. | | | | AIC: 3197.6 |
| Effect | | Adj. mean diff (95% CI), p | |  |
| Difference: AA vs. AG/GG | | 0.04 (-2.54, 2.62), p=0.9759 | |  |
| Changes over time:  Week 8 - Baseline  Week 16 - Baseline  Week 16 - Week 8 | | -2.45 (-3.50, -1.39), p<0.0001  -2.16 (-3.17, -1.15), p<0.0001  0.29 (-0.79, 1.36), p=0.6004 | |  |
| Model 2: fixed treatment effect difference by genotype. | | | | AIC: 3197.1 |
| Effect | | Adj. mean diff (95% CI), p | | Model Comparison |
| Difference: AA vs. AG/GG  At Baseline  Post-baseline | | 0.54 (-2.29, 3.37), p=0.7097  -0.25 (-2.93, 2.42), p=0.8521 | | 2 vs. 1: p=0.4041 |
| Changes over time (AG/GG):  Week 8 - Baseline  Week 16 - Baseline  Week 16 - Week 8 | | -1.91 (-3.56, -0.27), p=0.0232  -1.62 (-3.24, -0.01), p=0.0498  0.29 (-0.79, 1.37), p=0.5978 | |  |
| Changes over time (AA):  Week 8 - Baseline  Week 16 - Baseline  Week 16 - Week 8 | | -2.71 (-3.93, -1.48), p<0.0001  -2.42 (-3.59, -1.24), p=0.0001  0.29 (-0.79, 1.37), p=0.5978 | |  |
| Model 3: full model. Treatment effect difference by genotype, changing over time. | | | | AIC: 3190.7 |
| Effect | | Adj. mean diff (95% CI), p | | Model Comparison |
| Difference: AA vs. AG/GG  At Baseline  At Week 8  At Week 16 | | 0.54 (-2.29, 3.37), p=0.7094  1.33 (-1.61, 4.27), p=0.3773  -1.58 (-4.45, 1.28), p=0.2801 | | 3 vs. 1: p=0.0301  3 vs. 2 p=0.0120 |
| Changes over time (AG/GG):  Week 8 - Baseline  Week 16 - Baseline  Week 16 - Week 8 | | -2.98 (-4.80, -1.15), p=0.0016  -0.73 (-2.47, 1.02), p=0.4162  2.25 (0.39, 4.11), p=0.0187 | |  |
| Changes over time (AA):  Week 8 - Baseline  Week 16 - Baseline  Week 16 - Week 8 | | -2.19 (-3.46, -0.91), p=0.0009  -2.85 (-4.06, -1.64), p<0.0001  -0.66 (-1.97, 0.63), p=0.3188 | |  |

| **Table S6:** Secondary outcome: 24-hr ABPM nighttime mean SBP (mmHg) | | | | |
| --- | --- | --- | --- | --- |
|  |  | All | AA | AG/GG |
| Baseline | Nobs (Nmiss)  Mean (SD)  Median (Q1, Q3)  [Min, Max] | 174 (0)  127.0 (13.5)  125.5 (117.0, 134.0)  [91.0, 166.0] | 118 (0)  126.7 (13.4)  124.5 (118.0, 132.8)  [91.0, 166.0] | 56 (0)  127.6 (13.7)  126.5 (116.0, 137.0)  [105.0, 161.0] |
| Week 8 visit | Nobs (Nmiss)  Mean (SD)  Median (Q1, Q3)  [Min, Max] | 142 (32)  123.5 (14.8)  122.0 (114.0, 132.0)  [85.0, 160.0] | 95 (23)  124.1 (15.1)  122.0 (115.0, 135.0)  [85.0, 160.0] | 47 (9)  122.2 (14.2)  121.0 (112.5, 130.5)  [92.0, 154.0] |
| Change from baseline  to week 8 visit | Nobs (Nmiss)  Mean (SD)  Median (Q1, Q3)  [Min, Max] | 142 (32)  -4.1 (10.9)  -4.0 (-10.0, 4.0)  [-33.0, 25.0] | 95 (23)  -2.8 (11.1)  -2.0 (-10.0, 5.0)  [-30.0, 25.0] | 47 (9)  -6.5 (10.2)  -6.0 (-12.0, -1.0)  [-33.0, 18.0] |
| Week 16 visit | Nobs (Nmiss)  Mean (SD)  Median (Q1, Q3)  [Min, Max] | 162 (12)  122.5 (15.6)  121.0 (112.0, 132.0)  [93.0, 168.0] | 109 (9)  122.2 (15.0)  121.0 (112.0, 132.0)  [93.0, 156.0] | 53 (3)  123.2 (16.9)  122.0 (110.0, 134.0)  [94.0, 168.0] |
| Change from baseline  to week 16 visit | Nobs (Nmiss)  Mean (SD)  Median (Q1, Q3)  [Min, Max] | 162 (12)  -4.3 (13.1)  -3.0 (-12.0, 4.0)  [-37.0, 32.0] | 109 (9)  -4.5 (13.1)  -3.0 (-12.0, 4.0)  [-37.0, 26.0] | 53 (3)  -4.0 (13.2)  -3.0 (-12.0, 5.0)  [-37.0, 32.0] |
| Model 1: parallel lines. No difference in treatment effect by genotype. | | | | AIC: 3720.0 |
| Effect | | Adj. mean diff (95% CI), p | |  |
| Difference: AA vs. AG/GG | | -0.14 (-4.24, 3.96), p=0.9473 | |  |
| Changes over time:  Week 8 - Baseline  Week 16 - Baseline  Week 16 - Week 8 | | -3.87 (-5.79, -1.94), p=0.0001  -4.30 (-6.14, -2.47), p<0.0001  -0.44 (-2.40, 1.52), p=0.6633 | |  |
| Model 2: fixed treatment effect difference by genotype. | | | | AIC: 3718.6 |
| Effect | | Adj. mean diff (95% CI), p | | Model Comparison |
| Difference: AA vs. AG/GG  At Baseline  Post-baseline | | -0.87 (-5.49, 3.75), p=0.7134  0.29 (-3.99, 4.58), p=0.8935 | | 2 vs. 1: p=0.5029 |
| Changes over time (AG/GG):  Week 8 - Baseline  Week 16 - Baseline  Week 16 - Week 8 | | -4.65 (-7.64, -1.66), p=0.0026  -5.09 (-8.03, -2.15), p=0.0008  -0.44 (-2.40, 1.52), p=0.6618 | |  |
| Changes over time (AA):  Week 8 - Baseline  Week 16 - Baseline  Week 16 - Week 8 | | -3.49 (-5.71, -1.26), p=0.0023  -3.93 (-6.07, -1.78), p=0.0004  -0.44 (-2.40, 1.52), p=0.6618 | |  |
| Model 3: full model. Treatment effect difference by genotype, changing over time. | | | | AIC: 3714.3 |
| Effect | | Adj. mean diff (95% CI), p | | Model Comparison |
| Difference: AA vs. AG/GG  At Baseline  At Week 8  At Week 16 | | -0.87 (-5.48, 3.75), p=0.7134  2.27 (-2.57, 7.11), p=0.3598  -1.38 (-6.07, 3.31), p=0.5661 | | 3 vs. 1: p=0.1812  3 vs. 2 p=0.0849 |
| Changes over time (AG/GG):  Week 8 - Baseline  Week 16 - Baseline  Week 16 - Week 8 | | -5.97 (-9.30, -2.64), p=0.0005  -3.96 (-7.15, -0.77), p=0.0158  2.01 (-1.39, 5.40), p=0.2496 | |  |
| Changes over time (AA):  Week 8 - Baseline  Week 16 - Baseline  Week 16 - Week 8 | | -2.83 (-5.16, -0.50), p=0.0185  -4.47 (-6.69, -2.25), p=0.0001  -1.64 (-4.03, 0.74), p=0.1798 | |  |

| **Table S7:**  Secondary outcome: 24-hr ABPM nighttime mean DBP(mmHg) | | | | |
| --- | --- | --- | --- | --- |
|  |  | All | AA | AG/GG |
| Baseline | Nobs (Nmiss)  Mean (SD)  Median (Q1, Q3)  [Min, Max] | 174 (0)  73.3 (9.2)  74.0 (66.0, 79.0)  [48.0, 100.0] | 118 (0)  72.9 (9.3)  73.0 (66.0, 79.0)  [48.0, 100.0] | 56 (0)  74.0 (9.1)  75.0 (66.8, 81.2)  [52.0, 89.0] |
| Week 8 visit | Nobs (Nmiss)  Mean (SD)  Median (Q1, Q3)  [Min, Max] | 142 (32)  71.5 (9.0)  71.5 (67.0, 77.0)  [48.0, 100.0] | 95 (23)  71.6 (8.9)  71.0 (66.5, 76.5)  [51.0, 100.0] | 47 (9)  71.4 (9.3)  74.0 (67.5, 77.0)  [48.0, 91.0] |
| Change from baseline  to week 8 visit | Nobs (Nmiss)  Mean (SD)  Median (Q1, Q3)  [Min, Max] | 142 (32)  -2.0 (6.5)  -1.0 (-6.0, 2.0)  [-21.0, 16.0] | 95 (23)  -1.4 (6.7)  -1.0 (-5.0, 2.5)  [-21.0, 16.0] | 47 (9)  -3.2 (6.1)  -2.0 (-8.0, 1.5)  [-18.0, 9.0] |
| Week 16 visit | Nobs (Nmiss)  Mean (SD)  Median (Q1, Q3)  [Min, Max] | 162 (12)  71.5 (9.0)  71.0 (66.0, 76.0)  [52.0, 96.0] | 109 (9)  71.1 (8.9)  71.0 (66.0, 75.0)  [53.0, 94.0] | 53 (3)  72.3 (9.3)  73.0 (68.0, 78.0)  [52.0, 96.0] |
| Change from baseline  to week 16 visit | Nobs (Nmiss)  Mean (SD)  Median (Q1, Q3)  [Min, Max] | 162 (12)  -1.9 (8.0)  -2.0 (-7.0, 4.0)  [-28.0, 21.0] | 109 (9)  -1.9 (8.1)  -2.0 (-7.0, 4.0)  [-28.0, 21.0] | 53 (3)  -1.9 (7.9)  -2.0 (-7.0, 3.0)  [-19.0, 19.0] |
| Model 1: parallel lines. No difference in treatment effect by genotype. | | | | AIC: 3260.9 |
| Effect | | Adj. mean diff (95% CI), p | |  |
| Difference: AA vs. AG/GG | | -0.55 (-3.19, 2.08), p=0.6810 | |  |
| Changes over time:  Week 8 - Baseline  Week 16 - Baseline  Week 16 - Week 8 | | -1.92 (-3.07, -0.76), p=0.0013  -1.86 (-2.96, -0.75), p=0.0011  0.06 (-1.12, 1.24), p=0.9176 | |  |
| Model 2: fixed treatment effect difference by genotype. | | | | AIC: 3260.3 |
| Effect | | Adj. mean diff (95% CI), p | | Model Comparison |
| Difference: AA vs. AG/GG  At Baseline  Post-baseline | | -1.07 (-4.00, 1.86), p=0.4770  -0.25 (-2.99, 2.50), p=0.8586 | | 2 vs. 1: p=0.4336 |
| Changes over time (AG/GG):  Week 8 - Baseline  Week 16 - Baseline  Week 16 - Week 8 | | -2.47 (-4.26, -0.67), p=0.0077  -2.41 (-4.17, -0.64), p=0.0081  0.06 (-1.12, 1.24), p=0.9196 | |  |
| Changes over time (AA):  Week 8 - Baseline  Week 16 - Baseline  Week 16 - Week 8 | | -1.65 (-2.99, -0.31), p=0.0164  -1.59 (-2.88, -0.30), p=0.0164  0.06 (-1.12, 1.24), p=0.9196 | |  |
| Model 3: full model. Treatment effect difference by genotype, changing over time. | | | | AIC: 3258.1 |
| Effect | | Adj. mean diff (95% CI), p | | Model Comparison |
| Difference: AA vs. AG/GG  At Baseline  At Week 8  At Week 16 | | -1.07 (-4.00, 1.86), p=0.4771  0.71 (-2.34, 3.77), p=0.6487  -1.06 (-4.03, 1.91), p=0.4850 | | 3 vs. 1: p=0.2787  3 vs. 2 p=0.1635 |
| Changes over time (AG/GG):  Week 8 - Baseline  Week 16 - Baseline  Week 16 - Week 8 | | -3.11 (-5.12, -1.10), p=0.0027  -1.86 (-3.78, 0.06), p=0.0597  1.25 (-0.80, 3.30), p=0.2336 | |  |
| Changes over time (AA):  Week 8 - Baseline  Week 16 - Baseline  Week 16 - Week 8 | | -1.33 (-2.74, 0.08), p=0.0657  -1.85 (-3.19, -0.52), p=0.0071  -0.52 (-1.96, 0.91), p=0.4760 | |  |

| **Table S8:** Secondary outcome: Mean HBPM SBP (mmHg) | | | | |
| --- | --- | --- | --- | --- |
|  |  | All | AA | AG/GG |
| Screening | Nobs (Nmiss)  Mean (SD)  Median (Q1, Q3)  [Min, Max] | 96 (78)  143.8 (9.4)  143.3 (138.1, 148.0)  [123.2, 172.0] | 67 (51)  145.6 (9.5)  145.0 (138.6, 150.7)  [127.4, 172.0] | 29 (27)  139.9 (8.0)  139.1 (134.7, 143.1)  [123.2, 157.0] |
| Week 4 visit | Nobs (Nmiss)  Mean (SD)  Median (Q1, Q3)  [Min, Max] | 168 (6)  137.3 (12.0)  135.8 (128.9, 143.2)  [110.0, 176.4] | 114 (4)  137.5 (12.6)  135.5 (128.8, 143.4)  [110.0, 176.4] | 54 (2)  137.1 (10.6)  135.9 (129.2, 142.9)  [118.2, 163.0] |
| Change from screening  to week 4 visit | Nobs (Nmiss)  Mean (SD)  Median (Q1, Q3)  [Min, Max] | 90 (84)  -6.1 (10.0)  -5.4 (-12.6, -0.1)  [-28.7, 22.9] | 63 (55)  -7.3 (10.3)  -8.8 (-13.4, -1.4)  [-28.7, 22.9] | 27 (29)  -3.5 (9.0)  -3.1 (-6.5, 0.2)  [-26.2, 15.1] |
| Week 12 visit | Nobs (Nmiss)  Mean (SD)  Median (Q1, Q3)  [Min, Max] | 156 (18)  136.6 (11.4)  135.6 (127.9, 143.0)  [115.3, 171.4] | 105 (13)  137.8 (12.0)  136.9 (128.2, 145.7)  [115.5, 171.4] | 51 (5)  134.1 (9.6)  134.0 (127.3, 140.8)  [115.3, 159.3] |
| Change from screening  to week 12 visit | Nobs (Nmiss)  Mean (SD)  Median (Q1, Q3)  [Min, Max] | 85 (89)  -7.6 (10.3)  -6.5 (-16.1, -0.6)  [-31.3, 26.2] | 58 (60)  -8.3 (10.5)  -7.7 (-16.6, -1.8)  [-30.2, 26.2] | 27 (29)  -6.1 (9.8)  -4.8 (-11.8, 0.7)  [-31.3, 15.1] |
| Model 1: parallel lines. No difference in treatment effect by genotype. | | | | AIC: 3034.9 |
| Effect | | Adj. mean diff (95% CI), p | |  |
| Difference: AA vs. AG/GG | | 2.17 (-1.16, 5.48), p=0.2030 | |  |
| Changes over time:  Week 4 - Screening  Week 12 - Screening  Week 12 - Week 4 | | -6.46 (-8.07, -4.85), p<0.0001  -7.09 (-8.72, -5.46), p<0.0001  -0.63 (-1.95, 0.69), p=0.3493 | |  |
| Model 2: fixed treatment effect difference by genotype. | | | | AIC: 3031.1 |
| Effect | | Adj. mean diff (95% CI), p | | Model Comparison |
| Difference: AA vs. AG/GG  At Screening  Post-screening | | 4.42 (0.24, 8.60), p=0.0392  1.60 (-1.78, 4.97), p=0.3544 | | 2 vs. 1: p=0.0827 |
| Changes over time (AG/GG):  Week 4 - Screening  Week 12 - Screening  Week 12 - Week 4 | | -4.49 (-7.23, -1.75), p=0.0015  -5.14 (-7.88, -2.39), p=0.0003  -0.64 (-1.96, 0.67), p=0.3392 | |  |
| Changes over time (AA):  Week 4 - Screening  Week 12 - Screening  Week 12 - Week 4 | | -7.32 (-9.19, -5.45), p<0.0001  -7.96 (-9.86, -6.06), p<0.0001  -0.64 (-1.96, 0.67), p=0.3392 | |  |
| Model 3: full model. Treatment effect difference by genotype, changing over time. | | | | AIC: 3028.1 |
| Effect | | Adj. mean diff (95% CI), p | | Model Comparison |
| Difference: AA vs. AG/GG  At Screening  At Week 4  At Week 12 | | 4.45 (0.28, 8.61), p=0.0375  0.55 (-3.07, 4.16), p=0.7683  2.77 (-0.91, 6.43), p=0.1413 | | 3 vs. 1: p=0.0653  3 vs. 2 p=0.1179 |
| Changes over time (AG/GG):  Week 4 - Screening  Week 12 - Screening  Week 12 - Week 4 | | -3.75 (-6.63, -0.86), p=0.0119  -5.89 (-8.79, -3.00), p=0.0001  -2.14 (-4.44, 0.15), p=0.0694 | |  |
| Changes over time (AA):  Week 4 - Screening  Week 12 - Screening  Week 12 - Week 4 | | -7.66 (-9.57, -5.75), p<0.0001  -7.58 (-9.53, -5.63), p<0.0001  0.08 (-1.51, 1.67), p=0.9224 | |  |

| **Table S9:** Secondary outcome: Mean HBPM DBP (mmHg) | | | | |
| --- | --- | --- | --- | --- |
|  |  | All | AA | AG/GG |
| Screening | Nobs (Nmiss)  Mean (SD)  Median (Q1, Q3)  [Min, Max] | 97 (77)  87.4 (7.8)  87.9 (83.5, 92.1)  [65.6, 105.0] | 68 (50)  87.1 (8.4)  87.9 (83.1, 92.3)  [65.6, 105.0] | 29 (27)  88.1 (6.2)  88.3 (85.7, 91.4)  [76.2, 100.5] |
| Week 4 visit | Nobs (Nmiss)  Mean (SD)  Median (Q1, Q3)  [Min, Max] | 168 (6)  85.0 (8.2)  85.1 (80.6, 89.5)  [57.9, 107.9] | 114 (4)  84.6 (8.6)  84.6 (79.8, 89.4)  [61.2, 107.9] | 54 (2)  85.9 (7.3)  85.8 (83.3, 89.6)  [57.9, 103.8] |
| Change from screening  to week 4 visit | Nobs (Nmiss)  Mean (SD)  Median (Q1, Q3)  [Min, Max] | 91 (83)  -2.1 (5.8)  -2.8 (-4.8, 0.5)  [-17.4, 17.9] | 64 (54)  -2.6 (5.9)  -3.0 (-5.7, 0.1)  [-17.4, 17.9] | 27 (29)  -0.8 (5.3)  -0.7 (-4.1, 1.4)  [-12.6, 13.5] |
| Week 12 visit | Nobs (Nmiss)  Mean (SD)  Median (Q1, Q3)  [Min, Max] | 156 (18)  84.1 (7.2)  84.6 (79.8, 89.0)  [60.9, 103.1] | 105 (13)  84.0 (7.9)  84.4 (79.6, 89.4)  [60.9, 103.1] | 51 (5)  84.2 (5.6)  85.1 (81.2, 87.6)  [72.5, 94.7] |
| Change from screening  to week 12 visit | Nobs (Nmiss)  Mean (SD)  Median (Q1, Q3)  [Min, Max] | 85 (89)  -3.6 (5.0)  -3.1 (-7.2, -0.2)  [-19.6, 10.8] | 58 (60)  -3.8 (5.4)  -4.1 (-7.9, 0.6)  [-19.6, 7.2] | 27 (29)  -3.1 (4.2)  -3.0 (-7.1, -0.5)  [-9.1, 10.8] |
| Model 1: parallel lines. No difference in treatment effect by genotype. | | | | AIC: 2632.7 |
| Effect | | Adj. mean diff (95% CI), p | |  |
| Difference: AA vs. AG/GG | | -0.31 (-2.71, 2.09), p=0.7994 | |  |
| Changes over time:  Week 4 - Screening  Week 12 - Screening  Week 12 - Week 4 | | -2.35 (-3.23, -1.47), p<0.0001  -3.10 (-3.99, -2.21), p<0.0001  -0.75 (-1.47, -0.03), p=0.0414 | |  |
| Model 2: fixed treatment effect difference by genotype. | | | | AIC: 2632.1 |
| Effect | | Adj. mean diff (95% CI), p | | Model Comparison |
| Difference: AA vs. AG/GG  At Screening  Post-screening | | 0.42 (-2.36, 3.19), p=0.7695  -0.49 (-2.92, 1.93), p=0.6905 | | 2 vs. 1: p=0.3092 |
| Changes over time (AG/GG):  Week 4 - Screening  Week 12 - Screening  Week 12 - Week 4 | | -1.71 (-3.22, -0.20), p=0.0275  -2.47 (-3.98, -0.96), p=0.0016  -0.76 (-1.48, -0.04), p=0.0401 | |  |
| Changes over time (AA):  Week 4 - Screening  Week 12 - Screening  Week 12 - Week 4 | | -2.62 (-3.64, -1.60), p<0.0001  -3.38 (-4.42, -2.34), p<0.0001  -0.76 (-1.48, -0.04), p=0.0401 | |  |
| Model 3: full model. Treatment effect difference by genotype, changing over time. | | | | AIC: 2630.1 |
| Effect | | Adj. mean diff (95% CI), p | | Model Comparison |
| Difference: AA vs. AG/GG  At Screening  At Week 4  At Week 12 | | 0.43 (-2.33, 3.21), p=0.7593  -1.09 (-3.62, 1.43), p=0.3979  0.18 (-2.37, 2.73), p=0.8926 | | 3 vs. 1: p=0.1571  3 vs. 2 p=0.1024 |
| Changes over time (AG/GG):  Week 4 - Screening  Week 12 - Screening  Week 12 - Week 4 | | -1.28 (-2.87, 0.30), p=0.1155  -2.90 (-4.49, -1.31), p=0.0004  -1.62 (-2.87, -0.36), p=0.0126 | |  |
| Changes over time (AA):  Week 4 - Screening  Week 12 - Screening  Week 12 - Week 4 | | -2.81 (-3.86, -1.77), p<0.0001  -3.16 (-4.23, -2.09), p<0.0001  -0.35 (-1.21, 0.52), p=0.4377 | |  |

| **Table S10** Secondary outcome: Na^+^ (mmol/L) | | | | |
| --- | --- | --- | --- | --- |
|  |  | All | AA | AG/GG |
| Baseline | Nobs (Nmiss) Mean (SD) Median (Q1, Q3) [Min, Max] | 173 (1) 139.8 (2.3) 140.0 (139.0, 141.0) [130.0, 146.0] | 117 (1) 139.7 (2.3) 140.0 (139.0, 141.0) [130.0, 144.0] | 56 (0) 140.0 (2.4) 140.0 (139.0, 142.0) [133.0, 146.0] |
| Week 2 visit | Nobs (Nmiss) Mean (SD) Median (Q1, Q3) [Min, Max] | 171 (3) 139.6 (5.6) 140.0 (139.0, 141.0) [72.0, 145.0] | 116 (2) 139.8 (2.2) 140.0 (138.0, 141.0) [135.0, 145.0] | 55 (1) 139.2 (9.4) 141.0 (139.0, 142.0) [72.0, 144.0] |
| Change from baseline to week 2 visit | Nobs (Nmiss) Mean (SD) Median (Q1, Q3) [Min, Max] | 170 (4) -0.21 (5.76) 0.00 (-1.00, 1.75) [-69.00, 12.00] | 115 (3) 0.10 (2.20) 0.00 (-1.00, 1.00) [-4.00, 12.00] | 55 (1) -0.84 (9.64) 0.00 (-1.00, 2.00) [-69.00, 6.00] |
| Week 8 visit | Nobs (Nmiss) Mean (SD) Median (Q1, Q3) [Min, Max] | 171 (3) 139.9 (2.4) 140.0 (138.0, 141.0) [130.0, 147.0] | 115 (3) 139.8 (2.3) 140.0 (138.0, 141.0) [133.0, 147.0] | 56 (0) 140.1 (2.5) 140.0 (138.8, 141.0) [130.0, 147.0] |
| Change from baseline to week 8 visit | Nobs (Nmiss) Mean (SD) Median (Q1, Q3) [Min, Max] | 170 (4) 0.10 (2.29) 0.00 (-1.00, 1.00) [-7.00, 14.00] | 114 (4) 0.14 (2.23) 0.00 (-1.00, 1.00) [-4.00, 14.00] | 56 (0) 0.02 (2.44) 0.00 (-1.25, 1.00) [-7.00, 6.00] |
| Week 16 visit | Nobs (Nmiss) Mean (SD) Median (Q1, Q3) [Min, Max] | 164 (10) 140.0 (2.2) 140.0 (139.0, 142.0) [133.0, 146.0] | 110 (8) 139.9 (2.1) 140.0 (139.0, 141.0) [135.0, 145.0] | 54 (2) 140.2 (2.5) 140.5 (139.0, 142.0) [133.0, 146.0] |
| Change from baseline to week 16 visit | Nobs (Nmiss) Mean (SD) Median (Q1, Q3) [Min, Max] | 163 (11) 0.18 (2.21) 0.00 (-1.00, 1.00) [-5.00, 14.00] | 109 (9) 0.21 (2.22) 0.00 (-1.00, 1.00) [-4.00, 14.00] | 54 (2) 0.13 (2.20) 0.00 (-1.00, 2.00) [-5.00, 5.00] |
| Model 1: parallel lines. No difference in treatment effect by genotype. | | | | AIC: 3587.4 |
| Effect | | Adj. mean diff (95% CI), p | |  |
| Difference: AA vs. AG/GG | | -0.08 (-0.79, 0.62), p=0.8135 | |  |
| Changes over time: Week 2 - Baseline Week 8 - Baseline Week 16 - Baseline Week 8 - Week 2 Week 16 - Week 8 | | -0.21 (-0.86, 0.44), p=0.5339 0.11 (-0.54, 0.76), p=0.7377 0.18 (-0.48, 0.84), p=0.5954 0.32 (-0.33, 0.97), p=0.3404 0.07 (-0.59, 0.73), p=0.8417 | |  |
| Model 2: fixed treatment effect difference by genotype. | | | | AIC: 3588.3 |
| Effect | | Adj. mean diff (95% CI), p | | Model Comparison |
| Difference: AA vs. AG/GG At Baseline Post-baseline | | -0.36 (-1.46, 0.74), p=0.5218 0.01 (-0.75, 0.77), p=0.9811 | | 2 vs. 1: p=0.5225 |
| Changes over time (AG/GG): Week 2 - Baseline Week 8 - Baseline Week 16 - Baseline Week 8 - Week 2 Week 16 - Week 8 | | -0.46 (-1.46, 0.55), p=0.3748 -0.14 (-1.14, 0.87), p=0.7883 -0.07 (-1.08, 0.94), p=0.8915 0.32 (-0.33, 0.97), p=0.3396 0.07 (-0.59, 0.73), p=0.8413 | |  |
| Changes over time (AA): Week 2 - Baseline Week 8 - Baseline Week 16 - Baseline Week 8 - Week 2 Week 16 - Week 8 | | -0.09 (-0.83, 0.66), p=0.8190 0.23 (-0.52, 0.98), p=0.5451 0.30 (-0.46, 1.05), p=0.4387 0.32 (-0.33, 0.97), p=0.3396 0.07 (-0.59, 0.73), p=0.8413 | |  |
| Model 3: full model. Treatment effect difference by genotype, changing over time. | | | | AIC: 3588.5 |
| Effect | | Adj. mean diff (95% CI), p | | Model Comparison |
| Difference: AA vs. AG/GG At Baseline At Week 2 At Week 8 At Week 16 | | -0.36 (-1.46, 0.74), p=0.5219 0.55 (-0.56, 1.65), p=0.3340 -0.22 (-1.32, 0.88), p=0.6925 -0.30 (-1.42, 0.81), p=0.5954 | | 3 vs. 1: p=0.5423 3 vs. 2 p=0.4192 |
| Changes over time (AG/GG): Week 2 - Baseline Week 8 - Baseline Week 16 - Baseline Week 8 - Week 2 Week 16 - Week 8 | | -0.82 (-1.96, 0.32), p=0.1609 0.02 (-1.12, 1.15), p=0.9755 0.14 (-1.01, 1.29), p=0.8122 0.84 (-0.30, 1.98), p=0.1520 0.12 (-1.03, 1.27), p=0.8359 | |  |
| Changes over time (AA): Week 2 - Baseline Week 8 - Baseline Week 16 - Baseline Week 8 - Week 2 Week 16 - Week 8 | | 0.09 (-0.70, 0.87), p=0.8322 0.15 (-0.64, 0.94), p=0.7020 0.20 (-0.61, 1.00), p=0.6345 0.07 (-0.72, 0.86), p=0.8643 0.04 (-0.76, 0.84), p=0.9225 | |  |

| **Table S11** Secondary outcome: K^-^ (mmol/L) | | | | |
| --- | --- | --- | --- | --- |
|  |  | All | AA | AG/GG |
| Baseline | Nobs (Nmiss) Mean (SD) Median (Q1, Q3) [Min, Max] | 171 (3) 4.27 (0.31) 4.30 (4.10, 4.45) [3.50, 5.20] | 116 (2) 4.28 (0.29) 4.30 (4.10, 4.40) [3.50, 5.20] | 55 (1) 4.23 (0.34) 4.20 (4.00, 4.50) [3.60, 5.00] |
| Week 2 visit | Nobs (Nmiss) Mean (SD) Median (Q1, Q3) [Min, Max] | 169 (5) 4.23 (0.33) 4.20 (4.00, 4.40) [3.40, 5.20] | 115 (3) 4.25 (0.31) 4.20 (4.00, 4.40) [3.40, 5.20] | 54 (2) 4.21 (0.37) 4.20 (4.00, 4.47) [3.40, 5.10] |
| Change from baseline to week 2 visit | Nobs (Nmiss) Mean (SD) Median (Q1, Q3) [Min, Max] | 166 (8) -0.04 (0.31) 0.00 (-0.30, 0.20) [-0.80, 0.70] | 113 (5) -0.04 (0.32) 0.00 (-0.30, 0.20) [-0.80, 0.60] | 53 (3) -0.03 (0.29) 0.00 (-0.20, 0.10) [-0.70, 0.70] |
| Week 8 visit | Nobs (Nmiss) Mean (SD) Median (Q1, Q3) [Min, Max] | 171 (3) 4.22 (0.34) 4.20 (4.00, 4.40) [3.20, 5.70] | 115 (3) 4.23 (0.30) 4.20 (4.00, 4.40) [3.20, 5.10] | 56 (0) 4.19 (0.40) 4.20 (3.90, 4.40) [3.50, 5.70] |
| Change from baseline to week 8 visit | Nobs (Nmiss) Mean (SD) Median (Q1, Q3) [Min, Max] | 168 (6) -0.05 (0.33) -0.10 (-0.23, 0.10) [-0.80, 1.20] | 113 (5) -0.06 (0.30) -0.10 (-0.30, 0.20) [-0.70, 0.90] | 55 (1) -0.05 (0.37) -0.10 (-0.20, 0.10) [-0.80, 1.20] |
| Week 16 visit | Nobs (Nmiss) Mean (SD) Median (Q1, Q3) [Min, Max] | 163 (11) 4.22 (0.37) 4.20 (4.00, 4.40) [3.40, 5.70] | 109 (9) 4.25 (0.34) 4.20 (4.00, 4.40) [3.40, 5.70] | 54 (2) 4.16 (0.40) 4.20 (3.90, 4.38) [3.40, 5.10] |
| Change from baseline to week 16 visit | Nobs (Nmiss) Mean (SD) Median (Q1, Q3) [Min, Max] | 160 (14) -0.06 (0.29) -0.10 (-0.20, 0.10) [-0.70, 0.90] | 107 (11) -0.05 (0.29) -0.10 (-0.20, 0.10) [-0.70, 0.90] | 53 (3) -0.07 (0.29) 0.00 (-0.30, 0.10) [-0.60, 0.50] |
| Model 1: parallel lines. No difference in treatment effect by genotype. | | | | AIC: 209.8 |
| Effect | | Adj. mean diff (95% CI), p | |  |
| Difference: AA vs. AG/GG | | 0.06 (-0.03, 0.15), p=0.2033 | |  |
| Changes over time: Week 2 - Baseline Week 8 - Baseline Week 16 - Baseline Week 8 - Week 2 Week 16 - Week 8 | | -0.03 (-0.08, 0.01), p=0.1371 -0.05 (-0.10, -0.01), p=0.0234 -0.05 (-0.10, 0.00), p=0.0323 -0.02 (-0.06, 0.03), p=0.4380 0.00 (-0.04, 0.05), p=0.9260 | |  |
| Model 2: fixed treatment effect difference by genotype. | | | | AIC: 216.3 |
| Effect | | Adj. mean diff (95% CI), p | | Model Comparison |
| Difference: AA vs. AG/GG At Baseline Post-baseline | | 0.06 (-0.05, 0.16), p=0.3121 0.06 (-0.03, 0.15), p=0.2076 | | 2 vs. 1: p=0.9307 |
| Changes over time (AG/GG): Week 2 - Baseline Week 8 - Baseline Week 16 - Baseline Week 8 - Week 2 Week 16 - Week 8 | | -0.04 (-0.11, 0.03), p=0.3057 -0.06 (-0.13, 0.02), p=0.1264 -0.05 (-0.12, 0.02), p=0.1443 -0.02 (-0.06, 0.03), p=0.4388 0.00 (-0.04, 0.05), p=0.9260 | |  |
| Changes over time (AA): Week 2 - Baseline Week 8 - Baseline Week 16 - Baseline Week 8 - Week 2 Week 16 - Week 8 | | -0.03 (-0.09, 0.02), p=0.2098 -0.05 (-0.10, 0.00), p=0.0538 -0.05 (-0.10, 0.00), p=0.0682 -0.02 (-0.06, 0.03), p=0.4388 0.00 (-0.04, 0.05), p=0.9260 | |  |
| Model 3: full model. Treatment effect difference by genotype, changing over time. | | | | AIC: 228.2 |
| Effect | | Adj. mean diff (95% CI), p | | Model Comparison |
| Difference: AA vs. AG/GG At Baseline At Week 2 At Week 8 At Week 16 | | 0.06 (-0.05, 0.16), p=0.3123 0.05 (-0.06, 0.16), p=0.3711 0.05 (-0.06, 0.15), p=0.4091 0.08 (-0.02, 0.19), p=0.1319 | | 3 vs. 1: p=0.8723 3 vs. 2 p=0.7060 |
| Changes over time (AG/GG): Week 2 - Baseline Week 8 - Baseline Week 16 - Baseline Week 8 - Week 2 Week 16 - Week 8 | | -0.03 (-0.11, 0.05), p=0.4602 -0.05 (-0.13, 0.03), p=0.2608 -0.07 (-0.15, 0.01), p=0.0941 -0.02 (-0.10, 0.07), p=0.7071 -0.02 (-0.10, 0.06), p=0.5705 | |  |
| Changes over time (AA): Week 2 - Baseline Week 8 - Baseline Week 16 - Baseline Week 8 - Week 2 Week 16 - Week 8 | | -0.04 (-0.09, 0.02), p=0.1964 -0.06 (-0.11, 0.00), p=0.0481 -0.04 (-0.10, 0.02), p=0.1525 -0.02 (-0.08, 0.04), p=0.4928 0.01 (-0.04, 0.07), p=0.6075 | |  |

| **Table S12** Secondary outcome: Cl^-^ (mmol/L) | | | | |
| --- | --- | --- | --- | --- |
|  |  | All | AA | AG/GG |
| Baseline | Nobs (Nmiss) Mean (SD) Median (Q1, Q3) [Min, Max] | 95 (79) 104.8 (2.6) 105.0 (103.0, 107.0) [95.0, 110.0] | 68 (50) 104.7 (2.7) 105.0 (103.0, 107.0) [95.0, 110.0] | 27 (29) 105.2 (2.3) 105.0 (103.0, 106.5) [101.0, 109.0] |
| Week 2 visit | Nobs (Nmiss) Mean (SD) Median (Q1, Q3) [Min, Max] | 93 (81) 103.9 (2.2) 104.0 (103.0, 105.0) [99.0, 109.0] | 67 (51) 103.7 (2.4) 104.0 (102.0, 105.5) [99.0, 109.0] | 26 (30) 104.2 (1.8) 104.0 (103.0, 105.0) [101.0, 108.0] |
| Change from baseline to week 2 visit | Nobs (Nmiss) Mean (SD) Median (Q1, Q3) [Min, Max] | 92 (82) -0.96 (2.34) -1.00 (-2.25, 0.00) [-7.00, 8.00] | 66 (52) -0.95 (2.50) -1.00 (-2.00, 0.00) [-7.00, 8.00] | 26 (30) -0.96 (1.95) -1.00 (-2.75, 0.00) [-5.00, 3.00] |
| Week 8 visit | Nobs (Nmiss) Mean (SD) Median (Q1, Q3) [Min, Max] | 93 (81) 103.7 (2.3) 104.0 (102.0, 105.0) [98.0, 110.0] | 66 (52) 103.6 (2.5) 103.5 (102.0, 105.0) [98.0, 110.0] | 27 (29) 104.0 (1.7) 104.0 (103.0, 105.0) [101.0, 107.0] |
| Change from baseline to week 8 visit | Nobs (Nmiss) Mean (SD) Median (Q1, Q3) [Min, Max] | 92 (82) -1.12 (2.22) -1.00 (-3.00, 1.00) [-7.00, 5.00] | 65 (53) -1.08 (2.21) -1.00 (-3.00, 0.00) [-7.00, 5.00] | 27 (29) -1.22 (2.28) -2.00 (-2.50, 1.00) [-5.00, 4.00] |
| Week 16 visit | Nobs (Nmiss) Mean (SD) Median (Q1, Q3) [Min, Max] | 89 (85) 103.8 (2.3) 104.0 (102.0, 105.0) [97.0, 109.0] | 63 (55) 103.8 (2.4) 104.0 (102.0, 105.0) [99.0, 109.0] | 26 (30) 103.8 (2.2) 104.0 (103.0, 105.0) [97.0, 108.0] |
| Change from baseline to week 16 visit | Nobs (Nmiss) Mean (SD) Median (Q1, Q3) [Min, Max] | 88 (86) -1.01 (2.36) -1.00 (-2.00, 1.00) [-8.00, 7.00] | 62 (56) -0.89 (2.30) -1.00 (-2.00, 1.00) [-6.00, 7.00] | 26 (30) -1.31 (2.53) -1.00 (-2.75, 0.00) [-8.00, 4.00] |
| Model 1: parallel lines. No difference in treatment effect by genotype. | | | | AIC: 1559.8 |
| Effect | | Adj. mean diff (95% CI), p | |  |
| Difference: AA vs. AG/GG | | -0.31 (-1.18, 0.56), p=0.4911 | |  |
| Changes over time: Week 2 - Baseline Week 8 - Baseline Week 16 - Baseline Week 8 - Week 2 Week 16 - Week 8 | | -0.96 (-1.40, -0.52), p<0.0001 -1.10 (-1.54, -0.66), p<0.0001 -1.03 (-1.48, -0.58), p<0.0001 -0.14 (-0.58, 0.31), p=0.5468 0.07 (-0.38, 0.52), p=0.7731 | |  |
| Model 2: fixed treatment effect difference by genotype. | | | | AIC: 1561.4 |
| Effect | | Adj. mean diff (95% CI), p | | Model Comparison |
| Difference: AA vs. AG/GG At Baseline Post-baseline | | -0.47 (-1.52, 0.58), p=0.3836 -0.25 (-1.15, 0.64), p=0.5834 | | 2 vs. 1: p=0.5890 |
| Changes over time (AG/GG): Week 2 - Baseline Week 8 - Baseline Week 16 - Baseline Week 8 - Week 2 Week 16 - Week 8 | | -1.12 (-1.84, -0.40), p=0.0027 -1.25 (-1.97, -0.53), p=0.0008 -1.19 (-1.91, -0.46), p=0.0015 -0.14 (-0.58, 0.31), p=0.5498 0.07 (-0.38, 0.52), p=0.7729 | |  |
| Changes over time (AA): Week 2 - Baseline Week 8 - Baseline Week 16 - Baseline Week 8 - Week 2 Week 16 - Week 8 | | -0.90 (-1.39, -0.40), p=0.0005 -1.03 (-1.53, -0.54), p=0.0001 -0.97 (-1.47, -0.47), p=0.0002 -0.14 (-0.58, 0.31), p=0.5498 0.07 (-0.38, 0.52), p=0.7729 | |  |
| Model 3: full model. Treatment effect difference by genotype, changing over time. | | | | AIC: 1564.0 |
| Effect | | Adj. mean diff (95% CI), p | | Model Comparison |
| Difference: AA vs. AG/GG At Baseline At Week 2 At Week 8 At Week 16 | | -0.47 (-1.52, 0.58), p=0.3840 -0.45 (-1.51, 0.61), p=0.4061 -0.29 (-1.35, 0.76), p=0.5860 0.00 (-1.06, 1.06), p=0.9988 | | 3 vs. 1: p=0.7736 3 vs. 2 p=0.6629 |
| Changes over time (AG/GG): Week 2 - Baseline Week 8 - Baseline Week 16 - Baseline Week 8 - Week 2 Week 16 - Week 8 | | -0.97 (-1.80, -0.14), p=0.0233 -1.22 (-2.04, -0.40), p=0.0040 -1.36 (-2.19, -0.53), p=0.0015 -0.25 (-1.08, 0.58), p=0.5573 -0.14 (-0.97, 0.69), p=0.7407 | |  |
| Changes over time (AA): Week 2 - Baseline Week 8 - Baseline Week 16 - Baseline Week 8 - Week 2 Week 16 - Week 8 | | -0.95 (-1.47, -0.43), p=0.0004 -1.05 (-1.57, -0.52), p=0.0001 -0.89 (-1.42, -0.36), p=0.0012 -0.09 (-0.62, 0.43), p=0.7319 0.15 (-0.38, 0.69), p=0.5777 | |  |

| **Table S13** Secondary outcome: Creatinine (umol/L) | | | | |
| --- | --- | --- | --- | --- |
|  |  | All | AA | AG/GG |
| Baseline | Nobs (Nmiss) Mean (SD) Median (Q1, Q3) [Min, Max] | 173 (1) 72.2 (13.8) 72.0 (63.0, 80.0) [7.0, 109.0] | 117 (1) 70.3 (13.9) 70.0 (62.0, 78.0) [7.0, 109.0] | 56 (0) 76.2 (12.6) 77.0 (66.8, 85.2) [47.0, 106.0] |
| Week 2 visit | Nobs (Nmiss) Mean (SD) Median (Q1, Q3) [Min, Max] | 171 (3) 77.1 (13.2) 78.0 (68.0, 85.5) [47.0, 110.0] | 116 (2) 76.0 (13.2) 75.5 (67.0, 84.2) [47.0, 108.0] | 55 (1) 79.4 (13.2) 80.0 (70.0, 86.5) [53.0, 110.0] |
| Change from baseline to week 2 visit | Nobs (Nmiss) Mean (SD) Median (Q1, Q3) [Min, Max] | 170 (4) 4.72 (9.08) 3.00 (0.00, 8.00) [-12.00, 80.00] | 115 (3) 5.43 (10.23) 3.00 (0.50, 9.00) [-10.00, 80.00] | 55 (1) 3.24 (5.82) 3.00 (0.00, 6.00) [-12.00, 17.00] |
| Week 8 visit | Nobs (Nmiss) Mean (SD) Median (Q1, Q3) [Min, Max] | 171 (3) 76.8 (14.2) 76.0 (68.0, 86.0) [47.0, 124.0] | 115 (3) 75.5 (14.5) 75.0 (66.0, 83.5) [47.0, 124.0] | 56 (0) 79.5 (13.2) 77.5 (70.0, 89.0) [50.0, 110.0] |
| Change from baseline to week 8 visit | Nobs (Nmiss) Mean (SD) Median (Q1, Q3) [Min, Max] | 170 (4) 4.48 (8.34) 4.00 (1.00, 9.00) [-20.00, 65.00] | 114 (4) 5.06 (9.05) 4.00 (2.00, 9.00) [-20.00, 65.00] | 56 (0) 3.30 (6.57) 3.00 (-1.25, 8.00) [-10.00, 25.00] |
| Week 16 visit | Nobs (Nmiss) Mean (SD) Median (Q1, Q3) [Min, Max] | 164 (10) 76.6 (14.2) 74.0 (68.0, 84.2) [39.0, 116.0] | 110 (8) 75.5 (14.5) 73.0 (65.2, 84.0) [39.0, 116.0] | 54 (2) 79.1 (13.3) 78.5 (70.2, 86.5) [49.0, 113.0] |
| Change from baseline to week 16 visit | Nobs (Nmiss) Mean (SD) Median (Q1, Q3) [Min, Max] | 163 (11) 4.21 (9.52) 4.00 (0.00, 8.00) [-20.00, 65.00] | 109 (9) 5.21 (10.13) 4.00 (1.00, 9.00) [-17.00, 65.00] | 54 (2) 2.20 (7.86) 2.00 (-2.00, 6.00) [-20.00, 23.00] |
| Model 1: parallel lines. No difference in treatment effect by genotype. | | | | AIC: 4856.4 |
| Effect | | Adj. mean diff (95% CI), p | |  |
| Difference: AA vs. AG/GG | | -4.18 (-8.24, -0.12), p=0.0454 | |  |
| Changes over time: Week 2 - Baseline Week 8 - Baseline Week 16 - Baseline Week 8 - Week 2 Week 16 - Week 8 | | 4.72 (3.46, 5.98), p<0.0001 4.45 (3.19, 5.72), p<0.0001 4.19 (2.91, 5.47), p<0.0001 -0.27 (-1.54, 1.00), p=0.6801 -0.27 (-1.55, 1.02), p=0.6860 | |  |
| Model 2: fixed treatment effect difference by genotype. | | | | AIC: 4852.1 |
| Effect | | Adj. mean diff (95% CI), p | | Model Comparison |
| Difference: AA vs. AG/GG At Baseline Post-baseline | | -5.89 (-10.26, -1.51), p=0.0090 -3.59 (-7.69, 0.51), p=0.0879 | | 2 vs. 1: p=0.0401 |
| Changes over time (AG/GG): Week 2 - Baseline Week 8 - Baseline Week 16 - Baseline Week 8 - Week 2 Week 16 - Week 8 | | 3.17 (1.22, 5.11), p=0.0015 2.90 (0.96, 4.85), p=0.0036 2.64 (0.69, 4.59), p=0.0084 -0.26 (-1.53, 1.00), p=0.6849 -0.26 (-1.54, 1.02), p=0.6865 | |  |
| Changes over time (AA): Week 2 - Baseline Week 8 - Baseline Week 16 - Baseline Week 8 - Week 2 Week 16 - Week 8 | | 5.46 (4.02, 6.91), p<0.0001 5.20 (3.75, 6.65), p<0.0001 4.94 (3.47, 6.40), p<0.0001 -0.26 (-1.53, 1.00), p=0.6849 -0.26 (-1.54, 1.02), p=0.6865 | |  |
| Model 3: full model. Treatment effect difference by genotype, changing over time. | | | | AIC: 4850.7 |
| Effect | | Adj. mean diff (95% CI), p | | Model Comparison |
| Difference: AA vs. AG/GG At Baseline At Week 2 At Week 8 At Week 16 | | -5.89 (-10.26, -1.51), p=0.0090 -3.55 (-7.93, 0.83), p=0.1143 -4.19 (-8.56, 0.19), p=0.0626 -3.01 (-7.40, 1.39), p=0.1825 | | 3 vs. 1: p=0.1761 3 vs. 2 p=0.6947 |
| Changes over time (AG/GG): Week 2 - Baseline Week 8 - Baseline Week 16 - Baseline Week 8 - Week 2 Week 16 - Week 8 | | 3.14 (0.93, 5.35), p=0.0058 3.30 (1.10, 5.50), p=0.0035 2.25 (0.02, 4.47), p=0.0491 0.17 (-2.05, 2.38), p=0.8841 -1.05 (-3.28, 1.17), p=0.3553 | |  |
| Changes over time (AA): Week 2 - Baseline Week 8 - Baseline Week 16 - Baseline Week 8 - Week 2 Week 16 - Week 8 | | 5.48 (3.95, 7.00), p<0.0001 5.01 (3.47, 6.54), p<0.0001 5.13 (3.58, 6.69), p<0.0001 -0.47 (-2.01, 1.07), p=0.5498 0.13 (-1.44, 1.69), p=0.8757 | |  |

| **Table S14** Secondary outcome: eGFR (mL/min/1.73m^2^; 2012 CKD Epi formula, assuming non-black ethnicity) | | | | |
| --- | --- | --- | --- | --- |
|  |  | All | AA | AG/GG |
| Baseline | Nobs (Nmiss) Mean (SD) Median (Q1, Q3) [Min, Max] | 170 (4) 91.9 (17.1) 92.4 (82.5, 98.7) [63.5, 246.2] | 115 (3) 93.6 (18.5) 93.2 (84.5, 100.5) [64.2, 246.2] | 55 (1) 88.5 (13.2) 90.0 (77.8, 96.9) [63.5, 127.5] |
| Week 2 visit | Nobs (Nmiss) Mean (SD) Median (Q1, Q3) [Min, Max] | 168 (6) 86.9 (13.5) 86.6 (77.7, 96.3) [56.6, 127.5] | 114 (4) 87.4 (13.0) 86.6 (78.9, 97.1) [56.6, 120.3] | 54 (2) 85.9 (14.4) 85.3 (74.7, 95.1) [60.0, 127.5] |
| Change from baseline to week 2 visit | Nobs (Nmiss) Mean (SD) Median (Q1, Q3) [Min, Max] | 167 (7) -4.98 (14.39) -3.07 (-6.88, 0.00) [-164.80, 15.45] | 113 (5) -5.97 (16.95) -3.13 (-7.41, 0.00) [-164.80, 9.70] | 54 (2) -2.92 (5.89) -2.92 (-6.02, -0.15) [-19.54, 15.45] |
| Week 8 visit | Nobs (Nmiss) Mean (SD) Median (Q1, Q3) [Min, Max] | 168 (6) 86.8 (13.4) 86.8 (77.5, 96.8) [49.2, 128.2] | 113 (5) 87.7 (12.8) 88.1 (79.1, 96.9) [49.2, 118.8] | 55 (1) 85.1 (14.7) 84.3 (75.2, 95.4) [57.6, 128.2] |
| Change from baseline to week 8 visit | Nobs (Nmiss) Mean (SD) Median (Q1, Q3) [Min, Max] | 167 (7) -4.91 (13.29) -3.36 (-8.42, -0.56) [-151.73, 17.68] | 112 (6) -5.64 (15.38) -3.79 (-8.40, -1.00) [-151.73, 17.68] | 55 (1) -3.42 (7.27) -2.15 (-7.63, 1.17) [-32.01, 9.26] |
| Week 16 visit | Nobs (Nmiss) Mean (SD) Median (Q1, Q3) [Min, Max] | 162 (12) 87.2 (13.8) 88.5 (77.2, 96.1) [53.4, 125.2] | 109 (9) 87.8 (12.9) 89.4 (80.1, 96.2) [53.4, 119.5] | 53 (3) 86.1 (15.5) 84.8 (75.3, 95.6) [55.2, 125.2] |
| Change from baseline to week 16 visit | Nobs (Nmiss) Mean (SD) Median (Q1, Q3) [Min, Max] | 161 (13) -4.42 (14.32) -2.71 (-8.60, 0.00) [-151.73, 22.17] | 108 (10) -5.63 (16.42) -3.31 (-8.85, -0.58) [-151.73, 20.16] | 53 (3) -1.96 (8.19) -1.96 (-6.26, 1.48) [-23.30, 22.17] |
| Model 1: parallel lines. No difference in treatment effect by genotype. | | | | AIC: 5080.9 |
| Effect | | Adj. mean diff (95% CI), p | |  |
| Difference: AA vs. AG/GG | | 2.75 (-1.34, 6.84), p=0.1889 | |  |
| Changes over time: Week 2 - Baseline Week 8 - Baseline Week 16 - Baseline Week 8 - Week 2 Week 16 - Week 8 | | -4.97 (-6.70, -3.24), p<0.0001 -4.91 (-6.64, -3.17), p<0.0001 -4.42 (-6.18, -2.67), p<0.0001 0.06 (-1.68, 1.80), p=0.9436 0.48 (-1.28, 2.24), p=0.5902 | |  |
| Model 2: fixed treatment effect difference by genotype. | | | | AIC: 5076.4 |
| Effect | | Adj. mean diff (95% CI), p | | Model Comparison |
| Difference: AA vs. AG/GG At Baseline Post-baseline | | 4.98 (0.33, 9.64), p=0.0372 1.99 (-2.17, 6.15), p=0.3502 | | 2 vs. 1: p=0.0512 |
| Changes over time (AG/GG): Week 2 - Baseline Week 8 - Baseline Week 16 - Baseline Week 8 - Week 2 Week 16 - Week 8 | | -2.94 (-5.62, -0.27), p=0.0316 -2.89 (-5.55, -0.22), p=0.0347 -2.40 (-5.08, 0.28), p=0.0801 0.06 (-1.68, 1.79), p=0.9492 0.48 (-1.27, 2.24), p=0.5889 | |  |
| Changes over time (AA): Week 2 - Baseline Week 8 - Baseline Week 16 - Baseline Week 8 - Week 2 Week 16 - Week 8 | | -5.94 (-7.92, -3.96), p<0.0001 -5.88 (-7.87, -3.90), p<0.0001 -5.40 (-7.40, -3.40), p<0.0001 0.06 (-1.68, 1.79), p=0.9492 0.48 (-1.27, 2.24), p=0.5889 | |  |
| Model 3: full model. Treatment effect difference by genotype, changing over time. | | | | AIC: 5073.8 |
| Effect | | Adj. mean diff (95% CI), p | | Model Comparison |
| Difference: AA vs. AG/GG At Baseline At Week 2 At Week 8 At Week 16 | | 4.98 (0.33, 9.64), p=0.0373 1.75 (-2.92, 6.42), p=0.4646 2.79 (-1.87, 7.45), p=0.2433 1.40 (-3.30, 6.09), p=0.5611 | | 3 vs. 1: p=0.2227 3 vs. 2 p=0.7468 |
| Changes over time (AG/GG): Week 2 - Baseline Week 8 - Baseline Week 16 - Baseline Week 8 - Week 2 Week 16 - Week 8 | | -2.78 (-5.82, 0.26), p=0.0745 -3.42 (-6.44, -0.40), p=0.0273 -2.01 (-5.06, 1.05), p=0.2008 -0.64 (-3.68, 2.40), p=0.6798 1.42 (-1.64, 4.47), p=0.3655 | |  |
| Changes over time (AA): Week 2 - Baseline Week 8 - Baseline Week 16 - Baseline Week 8 - Week 2 Week 16 - Week 8 | | -6.02 (-8.11, -3.92), p<0.0001 -5.62 (-7.73, -3.52), p<0.0001 -5.59 (-7.72, -3.46), p<0.0001 0.40 (-1.71, 2.51), p=0.7140 0.03 (-2.11, 2.17), p=0.9797 | |  |

| **Table S15** Secondary outcome: eGFR (mL/min/1.73m^2^; 2021 CKD Epi formula, without ethnicity coefficient) | | | | |
| --- | --- | --- | --- | --- |
|  |  | All | AA | AG/GG |
| Baseline | Nobs (Nmiss) Mean (SD) Median (Q1, Q3) [Min, Max] | 170 (4) 95.8 (14.2) 96.7 (88.0, 103.2) [67.5, 200.1] | 115 (3) 97.3 (14.7) 97.9 (89.3, 104.3) [68.3, 200.1] | 55 (1) 92.7 (12.8) 94.2 (82.9, 101.3) [67.5, 129.1] |
| Week 2 visit | Nobs (Nmiss) Mean (SD) Median (Q1, Q3) [Min, Max] | 168 (6) 91.2 (13.2) 90.7 (81.9, 100.7) [59.1, 129.1] | 114 (4) 91.6 (12.8) 91.3 (83.2, 101.3) [59.1, 121.1] | 54 (2) 90.1 (14.1) 89.6 (79.2, 99.5) [63.9, 129.1] |
| Change from baseline to week 2 visit | Nobs (Nmiss) Mean (SD) Median (Q1, Q3) [Min, Max] | 167 (7) -4.59 (11.24) -2.96 (-6.75, 0.00) [-113.81, 16.03] | 113 (5) -5.41 (12.97) -3.13 (-7.14, 0.00) [-113.81, 9.68] | 54 (2) -2.87 (5.97) -2.62 (-6.28, -0.12) [-20.29, 16.03] |
| Week 8 visit | Nobs (Nmiss) Mean (SD) Median (Q1, Q3) [Min, Max] | 168 (6) 91.0 (13.2) 92.0 (82.0, 100.9) [52.8, 129.6] | 113 (5) 91.9 (12.6) 93.3 (84.8, 101.2) [52.8, 121.1] | 55 (1) 89.2 (14.5) 88.9 (79.3, 100.1) [60.9, 129.6] |
| Change from baseline to week 8 visit | Nobs (Nmiss) Mean (SD) Median (Q1, Q3) [Min, Max] | 167 (7) -4.59 (10.21) -3.30 (-8.34, -0.43) [-101.12, 17.44] | 112 (6) -5.12 (11.32) -3.63 (-8.33, -0.78) [-101.12, 17.44] | 55 (1) -3.49 (7.45) -1.65 (-7.95, 0.96) [-33.05, 9.36] |
| Week 16 visit | Nobs (Nmiss) Mean (SD) Median (Q1, Q3) [Min, Max] | 162 (12) 91.4 (13.6) 93.6 (82.1, 100.4) [57.2, 127.3] | 109 (9) 92.1 (12.7) 94.1 (83.1, 100.7) [57.2, 121.6] | 53 (3) 90.2 (15.3) 89.1 (79.2, 100.4) [58.6, 127.3] |
| Change from baseline to week 16 visit | Nobs (Nmiss) Mean (SD) Median (Q1, Q3) [Min, Max] | 161 (13) -4.06 (11.40) -2.34 (-7.87, 0.00) [-101.12, 23.15] | 108 (10) -5.06 (12.55) -2.69 (-8.95, -0.44) [-101.12, 20.99] | 53 (3) -2.03 (8.35) -1.64 (-6.60, 1.25) [-24.54, 23.15] |
| Model 1: parallel lines. No difference in treatment effect by genotype. | | | | AIC: 4904.7 |
| Effect | | Adj. mean diff (95% CI), p | |  |
| Difference: AA vs. AG/GG | | 2.73 (-1.19, 6.65), p=0.1744 | |  |
| Changes over time: Week 2 - Baseline Week 8 - Baseline Week 16 - Baseline Week 8 - Week 2 Week 16 - Week 8 | | -4.57 (-6.05, -3.10), p<0.0001 -4.57 (-6.05, -3.10), p<0.0001 -4.06 (-5.55, -2.57), p<0.0001 0.00 (-1.48, 1.48), p=1.0000 0.52 (-0.98, 2.01), p=0.4996 | |  |
| Model 2: fixed treatment effect difference by genotype. | | | | AIC: 4900.9 |
| Effect | | Adj. mean diff (95% CI), p | | Model Comparison |
| Difference: AA vs. AG/GG At Baseline Post-baseline | | 4.54 (0.18, 8.89), p=0.0424 2.11 (-1.86, 6.08), p=0.2998 | | 2 vs. 1: p=0.0627 |
| Changes over time (AG/GG): Week 2 - Baseline Week 8 - Baseline Week 16 - Baseline Week 8 - Week 2 Week 16 - Week 8 | | -2.93 (-5.20, -0.66), p=0.0118 -2.94 (-5.20, -0.67), p=0.0115 -2.42 (-4.70, -0.14), p=0.0380 -0.01 (-1.48, 1.47), p=0.9944 0.52 (-0.97, 2.00), p=0.4984 | |  |
| Changes over time (AA): Week 2 - Baseline Week 8 - Baseline Week 16 - Baseline Week 8 - Week 2 Week 16 - Week 8 | | -5.36 (-7.04, -3.68), p<0.0001 -5.36 (-7.05, -3.68), p<0.0001 -4.85 (-6.55, -3.15), p<0.0001 -0.01 (-1.48, 1.47), p=0.9944 0.52 (-0.97, 2.00), p=0.4984 | |  |
| Model 3: full model. Treatment effect difference by genotype, changing over time. | | | | AIC: 4898.7 |
| Effect | | Adj. mean diff (95% CI), p | | Model Comparison |
| Difference: AA vs. AG/GG At Baseline At Week 2 At Week 8 At Week 16 | | 4.54 (0.19, 8.89), p=0.0425 1.81 (-2.55, 6.17), p=0.4178 2.94 (-1.42, 7.29), p=0.1887 1.55 (-2.83, 5.93), p=0.4898 | | 3 vs. 1: p=0.2304 3 vs. 2 p=0.6566 |
| Changes over time (AG/GG): Week 2 - Baseline Week 8 - Baseline Week 16 - Baseline Week 8 - Week 2 Week 16 - Week 8 | | -2.73 (-5.31, -0.15), p=0.0395 -3.49 (-6.06, -0.93), p=0.0081 -2.05 (-4.64, 0.55), p=0.1246 -0.76 (-3.34, 1.82), p=0.5641 1.45 (-1.15, 4.04), p=0.2774 | |  |
| Changes over time (AA): Week 2 - Baseline Week 8 - Baseline Week 16 - Baseline Week 8 - Week 2 Week 16 - Week 8 | | -5.46 (-7.24, -3.67), p<0.0001 -5.09 (-6.88, -3.31), p<0.0001 -5.03 (-6.84, -3.22), p<0.0001 0.36 (-1.43, 2.15), p=0.6930 0.06 (-1.75, 1.88), p=0.9481 | |  |

| **Table S16** Lab measurements - Uric Acid (umol/L) - per protocol by genotype group | | | | |
| --- | --- | --- | --- | --- |
|  |  | All | AA | AG/GG |
|  |  | N = 174 | N = 118 | N = 56 |
| Baseline | Nobs (Nmiss) Mean (SD) Median (Q1, Q3) [Min, Max] | 165 (9) 272.4 (163.1) 333.0 (227.0, 387.0) [0.2, 591.0] | 113 (5) 269.3 (159.5) 325.0 (232.0, 379.0) [0.2, 591.0] | 52 (4) 279.1 (172.0) 346.5 (155.3, 406.0) [0.2, 487.0] |
| Week 2 | Nobs (Nmiss) Mean (SD) Median (Q1, Q3) [Min, Max] | 101 (73) 359.6 (134.2) 384.0 (322.0, 433.0) [0.3, 588.0] | 70 (48) 368.5 (119.8) 382.5 (323.8, 435.2) [0.3, 588.0] | 31 (25) 339.5 (162.5) 392.0 (322.5, 429.5) [0.3, 557.0] |
| Change from baseline to week 2 | Nobs (Nmiss) Mean (SD) Median (Q1, Q3) [Min, Max] | 96 (78) 37.5 (39.7) 39.0 (3.5, 63.0) [-78.0, 156.0] | 67 (51) 39.9 (42.6) 45.0 (11.0, 68.5) [-78.0, 156.0] | 29 (27) 31.9 (31.9) 36.0 (0.1, 46.0) [-31.0, 86.0] |
| Week 8 | Nobs (Nmiss) Mean (SD) Median (Q1, Q3) [Min, Max] | 136 (38) 361.3 (139.1) 387.5 (329.8, 446.2) [0.3, 623.0] | 91 (27) 363.0 (127.5) 373.0 (329.5, 445.5) [0.3, 623.0] | 45 (11) 357.8 (161.6) 391.0 (333.0, 448.0) [0.3, 587.0] |
| Change from baseline to week 8 | Nobs (Nmiss) Mean (SD) Median (Q1, Q3) [Min, Max] | 129 (45) 37.8 (41.8) 41.0 (0.1, 59.0) [-72.0, 145.0] | 88 (30) 37.4 (42.3) 39.0 (0.1, 63.0) [-69.0, 139.0] | 41 (15) 38.6 (41.1) 42.0 (16.0, 56.0) [-72.0, 145.0] |
| Week 16 | Nobs (Nmiss) Mean (SD) Median (Q1, Q3) [Min, Max] | 160 (14) 296.6 (183.6) 367.5 (136.6, 429.2) [0.2, 553.0] | 108 (10) 292.1 (180.3) 358.5 (181.6, 428.2) [0.2, 553.0] | 52 (4) 306.0 (191.6) 388.5 (136.6, 442.5) [0.2, 551.0] |
| Change from baseline to week 16 | Nobs (Nmiss) Mean (SD) Median (Q1, Q3) [Min, Max] | 152 (22) 27.0 (42.3) 17.5 (0.0, 55.2) [-119.0, 165.0] | 103 (15) 27.7 (38.7) 18.0 (0.0, 56.5) [-46.0, 130.0] | 49 (7) 25.3 (49.4) 17.0 (0.0, 54.0) [-119.0, 165.0] |

| **Table S17** Characteristics of Adverse events | | | | | |
| --- | --- | --- | --- | --- | --- |
|  |  | All | AA | AG/GG | Missing |
|  |  | N = 94 | N = 53 | N = 35 | N = 6 |
| Related | Nobs (Nmiss) N (%) No N (%) Possible N (%) Probably N (%) Definite | 94 (0) 53 (56.4%) 35 (37.2%) 3 (3.2%) 3 (3.2%) | 53 (0) 33 (62.3%) 16 (30.2%) 2 (3.8%) 2 (3.8%) | 35 (0) 17 (48.6%) 16 (45.7%) 1 (2.9%) 1 (2.9%) | 6 (0) 3 (50.0%) 3 (50.0%) 0 (0.0%) 0 (0.0%) |
| Serious | Nobs (Nmiss) N (%) No N (%) Yes | 92 (2) 89 (96.7%) 3 (3.3%) | 52 (1) 50 (96.2%) 2 (3.8%) | 34 (1) 33 (97.1%) 1 (2.9%) | 6 (0) 6 (100.0%) 0 (0.0%) |

| **Table S18** Number of participants with at least one AE by MedDRA system organ class and preferred term, by genotype group (all participants in Full Analysis Set). | | | | | |
| --- | --- | --- | --- | --- | --- |
|  |  | All | AA | AG/GG | Missing |
| Number of randomised participants | | 222 | 131 | 65 | 26 |
| Number of participants with any event | | 48 | 28 | 16 | 4 |
| Cardiac disorders | | 3 (1.4%) | 1 (0.8%) | 2 (3.1%) | 2 (0.0%) |
|  | Acute myocardial infarction | 1 (0.5%) | 1 (0.8%) | 0 (0.0%) | 0 (0.0%) |
|  | Aortic valve incompetence | 1 (0.5%) | 0 (0.0%) | 1 (1.5%) | 1 (0.0%) |
|  | Extrasystoles | 1 (0.5%) | 0 (0.0%) | 1 (1.5%) | 1 (0.0%) |
| Ear and labyrinth disorders | | 1 (0.5%) | 1 (0.8%) | 0 (0.0%) | 0 (0.0%) |
|  | Tinnitus | 1 (0.5%) | 1 (0.8%) | 0 (0.0%) | 0 (0.0%) |
| Gastrointestinal disorders | | 10 (4.5%) | 7 (5.3%) | 2 (3.1%) | 2 (3.8%) |
|  | Abdominal discomfort | 1 (0.5%) | 1 (0.8%) | 0 (0.0%) | 0 (0.0%) |
|  | Abdominal pain upper | 1 (0.5%) | 1 (0.8%) | 0 (0.0%) | 0 (0.0%) |
|  | Constipation | 2 (0.9%) | 2 (1.5%) | 0 (0.0%) | 0 (0.0%) |
|  | Diarrhoea | 2 (0.9%) | 0 (0.0%) | 1 (1.5%) | 1 (3.8%) |
|  | Dry mouth | 2 (0.9%) | 1 (0.8%) | 1 (1.5%) | 1 (0.0%) |
|  | Nausea | 1 (0.5%) | 1 (0.8%) | 0 (0.0%) | 0 (0.0%) |
|  | Toothache | 1 (0.5%) | 1 (0.8%) | 0 (0.0%) | 0 (0.0%) |
| General disorders and administration site conditions | | 6 (2.7%) | 4 (3.1%) | 1 (1.5%) | 1 (3.8%) |
|  | Chest discomfort | 1 (0.5%) | 0 (0.0%) | 1 (1.5%) | 1 (0.0%) |
|  | Fatigue | 2 (0.9%) | 1 (0.8%) | 0 (0.0%) | 0 (3.8%) |
|  | Influenza like illness | 1 (0.5%) | 1 (0.8%) | 0 (0.0%) | 0 (0.0%) |
|  | Malaise | 1 (0.5%) | 1 (0.8%) | 0 (0.0%) | 0 (0.0%) |
|  | Night sweats | 1 (0.5%) | 1 (0.8%) | 0 (0.0%) | 0 (0.0%) |
| Infections and infestations | | 10 (4.5%) | 8 (6.1%) | 1 (1.5%) | 1 (3.8%) |
|  | Influenza | 1 (0.5%) | 1 (0.8%) | 0 (0.0%) | 0 (0.0%) |
|  | Rhinitis | 7 (3.2%) | 5 (3.8%) | 1 (1.5%) | 1 (3.8%) |
|  | Skin infection | 1 (0.5%) | 1 (0.8%) | 0 (0.0%) | 0 (0.0%) |
|  | Tonsillitis | 1 (0.5%) | 1 (0.8%) | 0 (0.0%) | 0 (0.0%) |
|  | Upper respiratory tract infection | 1 (0.5%) | 1 (0.8%) | 0 (0.0%) | 0 (0.0%) |
|  | Urinary tract infection | 1 (0.5%) | 1 (0.8%) | 0 (0.0%) | 0 (0.0%) |
|  | Viral infection | 1 (0.5%) | 1 (0.8%) | 0 (0.0%) | 0 (0.0%) |
| Injury, poisoning and procedural complications | | 1 (0.5%) | 1 (0.8%) | 0 (0.0%) | 0 (0.0%) |
|  | Fall | 1 (0.5%) | 1 (0.8%) | 0 (0.0%) | 0 (0.0%) |
| Investigations | | 3 (1.4%) | 1 (0.8%) | 2 (3.1%) | 2 (0.0%) |
|  | Blood sodium decreased | 1 (0.5%) | 0 (0.0%) | 1 (1.5%) | 1 (0.0%) |
|  | Blood uric acid increased | 2 (0.9%) | 1 (0.8%) | 1 (1.5%) | 1 (0.0%) |
| Metabolism and nutrition disorders | | 1 (0.5%) | 1 (0.8%) | 0 (0.0%) | 0 (0.0%) |
|  | Decreased appetite | 1 (0.5%) | 1 (0.8%) | 0 (0.0%) | 0 (0.0%) |
| Musculoskeletal and connective tissue disorders | | 5 (2.3%) | 1 (0.8%) | 3 (4.6%) | 3 (3.8%) |
|  | Arthralgia | 1 (0.5%) | 0 (0.0%) | 1 (1.5%) | 1 (0.0%) |
|  | Back pain | 3 (1.4%) | 0 (0.0%) | 2 (3.1%) | 2 (3.8%) |
|  | Joint swelling | 1 (0.5%) | 1 (0.8%) | 0 (0.0%) | 0 (0.0%) |
| Nervous system disorders | | 12 (5.4%) | 6 (4.6%) | 6 (9.2%) | 6 (0.0%) |
|  | Dizziness | 4 (1.8%) | 2 (1.5%) | 2 (3.1%) | 2 (0.0%) |
|  | Dizziness postural | 1 (0.5%) | 1 (0.8%) | 0 (0.0%) | 0 (0.0%) |
|  | Headache | 1 (0.5%) | 0 (0.0%) | 1 (1.5%) | 1 (0.0%) |
|  | Hypoaesthesia | 1 (0.5%) | 0 (0.0%) | 1 (1.5%) | 1 (0.0%) |
|  | Lethargy | 5 (2.3%) | 2 (1.5%) | 3 (4.6%) | 3 (0.0%) |
|  | Presyncope | 1 (0.5%) | 1 (0.8%) | 0 (0.0%) | 0 (0.0%) |
| Psychiatric disorders | | 2 (0.9%) | 1 (0.8%) | 1 (1.5%) | 1 (0.0%) |
|  | Anxiety | 1 (0.5%) | 1 (0.8%) | 0 (0.0%) | 0 (0.0%) |
|  | Insomnia | 1 (0.5%) | 0 (0.0%) | 1 (1.5%) | 1 (0.0%) |
| Reproductive system and breast disorders | | 1 (0.5%) | 0 (0.0%) | 0 (0.0%) | 0 (3.8%) |
|  | Erectile dysfunction | 1 (0.5%) | 0 (0.0%) | 0 (0.0%) | 0 (3.8%) |
| Respiratory, thoracic and mediastinal disorders | | 3 (1.4%) | 2 (1.5%) | 1 (1.5%) | 1 (0.0%) |
|  | Dyspnoea | 1 (0.5%) | 1 (0.8%) | 0 (0.0%) | 0 (0.0%) |
|  | Haemoptysis | 1 (0.5%) | 1 (0.8%) | 0 (0.0%) | 0 (0.0%) |
|  | Nasal congestion | 1 (0.5%) | 0 (0.0%) | 1 (1.5%) | 1 (0.0%) |
| Surgical and medical procedures | | 1 (0.5%) | 1 (0.8%) | 0 (0.0%) | 0 (0.0%) |
|  | Skin cyst excision | 1 (0.5%) | 1 (0.8%) | 0 (0.0%) | 0 (0.0%) |
| Vascular disorders | | 2 (0.9%) | 2 (1.5%) | 0 (0.0%) | 0 (0.0%) |
|  | Hot flush | 1 (0.5%) | 1 (0.8%) | 0 (0.0%) | 0 (0.0%) |
|  | Orthostatic hypotension | 1 (0.5%) | 1 (0.8%) | 0 (0.0%) | 0 (0.0%) |

| **Table S19** Withdrawals and withdrawal reasons – Full Analysis Set | | | | | |
| --- | --- | --- | --- | --- | --- |
|  |  | All | AA | AG/GG | Missing |
|  |  | N = 222 | N = 131 | N = 65 | N = 26 |
| Withdrawn | Nobs (Nmiss) N (%) Not Withdrawn N (%) Withdrawn | 222 (0) 182 (82.0%) 40 (18.0%) | 131 (0) 118 (90.1%) 13 (9.9%) | 65 (0) 54 (83.1%) 11 (16.9%) | 26 (0) 10 (38.5%) 16 (61.5%) |
| Withdrawal reasons for withdrawals prior to week 16 visit | Nobs (Nmiss) N (%) Adverse Event N (%) Physician recommendation N (%) Participans decision N (%) Other - please specify | 34 (0) 2 (5.9%) 17 (50.0%) 9 (26.5%) 6 (17.6%) | 11 (0) 1 (9.1%) 7 (63.6%) 3 (27.3%) 0 (0.0%) | 8 (0) 0 (0.0%) 4 (50.0%) 1 (12.5%) 3 (37.5%) | 15 (0) 1 (6.7%) 6 (40.0%) 5 (33.3%) 3 (20.0%) |
| Withdrawal reasons for withdrawals after attending week 16 visit | Nobs (Nmiss) N (%) Adverse Event N (%) Physician recommendation N (%) Participants decision N (%) Other - please specify | 6 (0) 1 (16.7%) 4 (66.7%) 0 (0.0%) 1 (16.7%) | 2 (0) 0 (0.0%) 1 (50.0%) 0 (0.0%) 1 (50.0%) | 3 (0) 1 (33.3%) 2 (66.7%) 0 (0.0%) 0 (0.0%) | 1 (0) 0 (0.0%) 1 (100.0%) 0 (0.0%) 0 (0.0%) |

| **Table S20** Medication stopping – Full Analysis Set | | | | | |
| --- | --- | --- | --- | --- | --- |
|  |  | All | AA | AG/GG | Missing |
|  |  | N = 222 | N = 131 | N = 65 | N = 26 |
| Medication stopped | Nobs (Nmiss) N (%) Yes N (%) No | 200 (22) 41 (20.5%) 159 (79.5%) | 127 (4) 27 (21.3%) 100 (78.7%) | 59 (6) 13 (22.0%) 46 (78.0%) | 14 (12) 1 (7.1%) 13 (92.9%) |
| Medication interrupted | Nobs (Nmiss) N (%) Yes N (%) No | 185 (37) 90 (48.6%) 95 (51.4%) | 121 (10) 59 (48.8%) 62 (51.2%) | 58 (7) 29 (50.0%) 29 (50.0%) | 6 (20) 2 (33.3%) 4 (66.7%) |

| **Table S21** Concomitant medications of special interest – Full Analysis Set | | | | | |
| --- | --- | --- | --- | --- | --- |
|  |  | All | AA | AG/GG | Missing |
|  |  | N = 222 | N = 131 | N = 65 | N = 26 |
| Aminoglycosides | Nobs (Nmiss) N (%) Yes N (%) No | 222 (0) 0 (0.0%) 222 (100.0%) | 131 (0) 0 (0.0%) 131 (100.0%) | 65 (0) 0 (0.0%) 65 (100.0%) | 26 (0) 0 (0.0%) 26 (100.0%) |
| Cephalosporins | Nobs (Nmiss) N (%) Yes N (%) No | 222 (0) 0 (0.0%) 222 (100.0%) | 131 (0) 0 (0.0%) 131 (100.0%) | 65 (0) 0 (0.0%) 65 (100.0%) | 26 (0) 0 (0.0%) 26 (100.0%) |
| Theophylline | Nobs (Nmiss) N (%) Yes N (%) No | 222 (0) 0 (0.0%) 222 (100.0%) | 131 (0) 0 (0.0%) 131 (100.0%) | 65 (0) 0 (0.0%) 65 (100.0%) | 26 (0) 0 (0.0%) 26 (100.0%) |
| NSAIDs | Nobs (Nmiss) N (%) Yes N (%) No | 222 (0) 13 (5.9%) 209 (94.1%) | 131 (0) 8 (6.1%) 123 (93.9%) | 65 (0) 5 (7.7%) 60 (92.3%) | 26 (0) 0 (0.0%) 26 (100.0%) |
| Digoxin | Nobs (Nmiss) N (%) Yes N (%) No | 222 (0) 1 (0.5%) 221 (99.5%) | 131 (0) 0 (0.0%) 131 (100.0%) | 65 (0) 1 (1.5%) 64 (98.5%) | 26 (0) 0 (0.0%) 26 (100.0%) |
| Sulphonylureas | Nobs (Nmiss) N (%) Yes N (%) No | 222 (0) 0 (0.0%) 222 (100.0%) | 131 (0) 0 (0.0%) 131 (100.0%) | 65 (0) 0 (0.0%) 65 (100.0%) | 26 (0) 0 (0.0%) 26 (100.0%) |
| Other antihypertensive medications | Nobs (Nmiss) N (%) Yes N (%) No | 222 (0) 204 (91.9%) 18 (8.1%) | 131 (0) 124 (94.7%) 7 (5.3%) | 65 (0) 61 (93.8%) 4 (6.2%) | 26 (0) 19 (73.1%) 7 (26.9%) |
| NSAIDs ongoing | Nobs (Nmiss) N (%) Yes N (%) No | 13 (0) 9 (69.2%) 4 (30.8%) | 8 (0) 5 (62.5%) 3 (37.5%) | 5 (0) 4 (80.0%) 1 (20.0%) | 0 (0) 0 (-%) 0 (-%) |
| Maximum duration of finished courses of NSAIDs | Nobs (Nmiss) Mean (SD) Median (Q1, Q3) [Min, Max] | 4 (9) 986.5 (1103.3) 734.0 (235.5, 1485.0) [6.0, 2472.0] | 3 (5) 1313.3 (1088.6) 1156.0 (734.0, 1814.0) [312.0, 2472.0] | 1 (4) 6.0 (-) 6.0 (6.0, 6.0) [6.0, 6.0] | 0 (0) - (-) - (-, -) [-, -] |

**Figure S1:**  Post Hoc Analyses of Daytime Systolic Blood Pressure Measurements Over Time. This figure presents the results of additional post hoc analyses, modelling all available daytime SBP measurements (from Ambulatory Blood Pressure Monitoring, Home Blood Pressure Monitoring, and Office Blood Pressure measurements) recorded at or after baseline. The analyses model daytime SBP as a function of time since baseline for two genotype groups (AA and AG/GG). Panel A (AA Genotype): This panel shows the model-predicted daytime SBP over time for participants with the AA genotype. The black line represents the predicted SBP, while the shaded area indicates the 95% confidence bands. Coloured dots and lines overlay the observed mean daytime SBP values at each study visit, plotted against the mean time from baseline of the corresponding measurements. Panel B (AG/GG Genotypes): Similar to Panel A, this panel displays the model-predicted daytime SBP for participants with the AG/GG genotypes, with the black line for predicted SBP and shaded area for 95% confidence bands. Observed mean SBP values are overlaid in colour. Panel C: This panel presents the model-predicted daytime SBP for both AA and AG/GG genotypes over time, with 95% confidence bands. It allows for a direct comparison of the predicted SBP between the two genotype groups. Panel D: This panel shows the change in model-predicted daytime SBP from baseline for both genotype groups, including 95% confidence bands, highlighting how SBP changes relative to baseline over time. Panel E: This panel illustrates the model-predicted differences in daytime SBP between the AA and AG/GG genotype groups, with 95% confidence bands, providing insight into the magnitude of SBP differences attributable to genotype. Panel F: This panel displays the model-predicted differences in the change in SBP from baseline between the AA and AG/GG genotype groups, with 95% confidence bands, showing how the change in SBP from baseline differs between the genotypes.


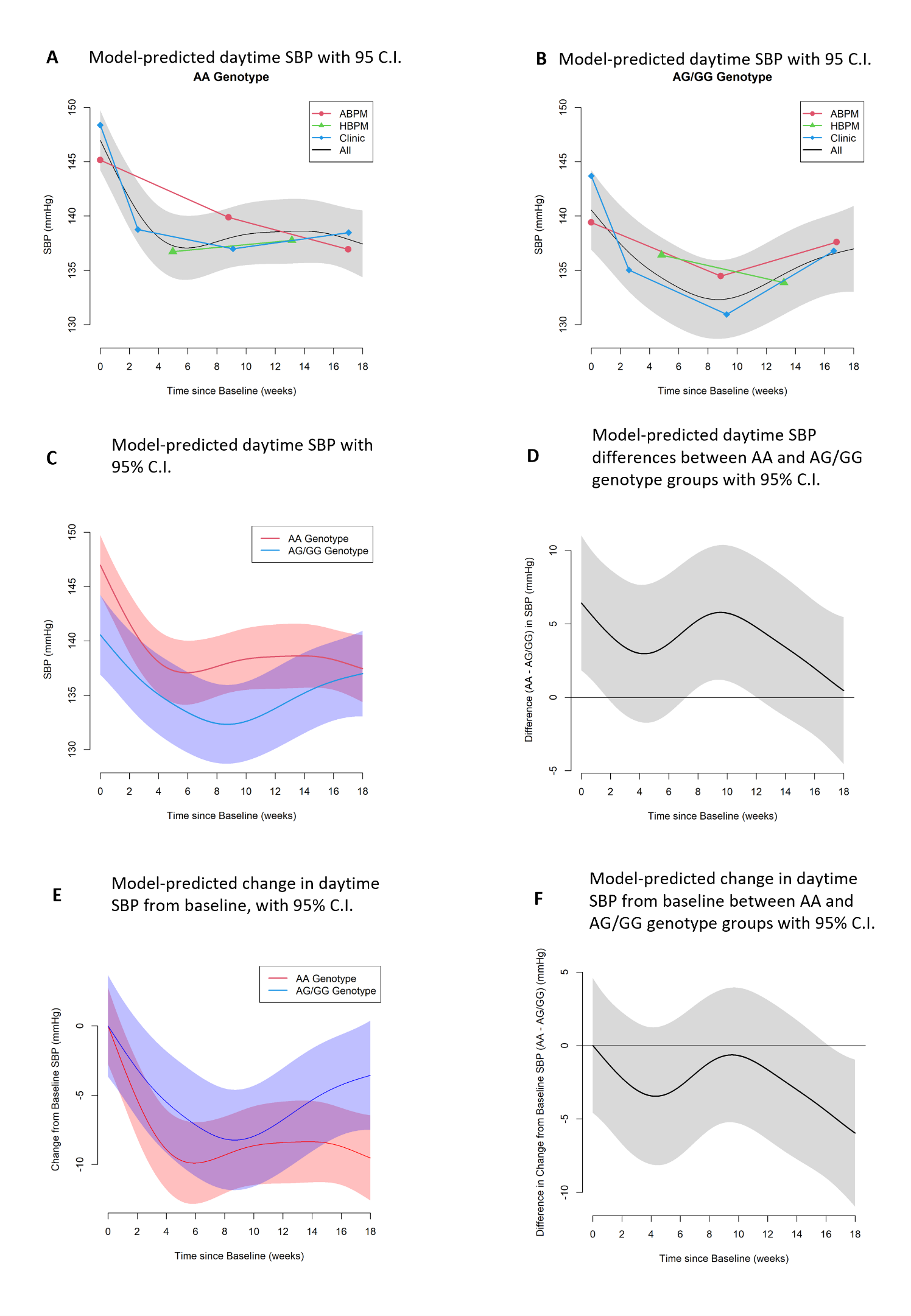

Supplement: Supplementary file 1 [file hyp-81-2049-s001.docx]
